# Defining Neighbourhoods as a Measure of Exposure to the Food Environment

**DOI:** 10.3390/ijerph120708504

**Published:** 2015-07-21

**Authors:** Anders K. Lyseen, Henning S. Hansen, Henrik Harder, Anders S. Jensen, Bent E. Mikkelsen

**Affiliations:** 1 Department of Development and Planning, Aalborg University, Copenhagen, A.C. Meyers Vænge 15, 2450 Copenhagen, Denmark; E-Mail: hsh@plan.aau.dk; 2 Department of Architecture, Design and Media technology, Aalborg University, Rendsburggade 14, 9000 Aalborg, Denmark; E-Mail: hhar@create.aau.dk; 3 LE34, Energivej 34, 2750 Ballerup, Denmark; E-Mail: asj@le34.dk; 4 Department of Clinical Medicine, Aalborg University, Copenhagen, A.C. Meyers Vænge 15, 2450 Copenhagen, Denmark; E-Mail: bemi@plan.aau.dk

**Keywords:** food environment, neighbourhood, exposure assessment, geographic information systems, Global Positioning System, activity spaces

## Abstract

Neighbourhoods are frequently used as a measure for individuals’ exposure to the food environment. However, the definitions of neighbourhoods fluctuate and have not been applied consistently in previous studies. Neighbourhoods defined from a single fixed location fail to capture people’s complete exposure in multiple locations, but measuring behaviour using traditional methods can be challenging. This study compares the traditional methods of measuring exposure to the food environment to methods that use data from GPS tracking. For each of the 187 participants, 11 different neighbourhoods were created in which the exposure to supermarkets and fast food outlets were measured. ANOVA, Tukey’s Honestly Significant Difference (HSD) test and *t*-tests were performed to compare the neighbourhoods. Significant differences were found between area sizes and the exposure to supermarkets and fast food outlets for different neighbourhood types. Second, significant differences in exposure to food outlets were found between the urban and rural neighbourhoods. Neighbourhoods are clearly a diffused and blurred concept that varies in meaning depending on each person’s perception and the conducted study. Complexity and heterogeneity of human mobility no longer appear to correspond to the use of residential neighbourhoods but rather emphasise the need for methods, concepts and measures of individual activity and exposure.

## 1. Introduction

Studies of nutrition and physical activity behaviour in the past decade have recognised the importance of the environment in understanding health and health related behaviour [1,2,3,4]. Within nutritional research, an increased focus has been placed on measuring the impact of the food environment on health outcomes such as Body Mass Index (BMI) [5,6,7,8], body weight [9,10], obesity [11,12] and diet [3,10,13]. The environmental exposure is often conceptualised through and measured within neighbourhoods. However, the spatial extent of neighbourhoods has proven difficult for researchers to define, and the result is a great variation in the definitions of neighbourhood used to study the environmental exposure [2].

The method used to define a neighbourhood is essential for researchers to ensure that measured exposure reaches optimal agreement with the actual exposure. However, for researchers to achieve this result, they must scrutinise the behaviour carefully to fully understand the phenomenon. The way a neighbourhood is defined should reflect the context of its application [14]. Therefore, when measuring the food environment, researchers must make qualified assumptions about where people shop or dine, the distance people are willing to travel for shopping or dining and other individual preferences [2].

Applying neighbourhoods to measuring food exposure creates a manageable concept to analyse the effect of the exposure. However, variations in neighbourhood definitions indicate that not all definitions manage to conceive and measure the actual exposure equally well [3,15]. Giles-Corti *et al.* found little agreement among previous studies on the appropriate distance from home, work or school to search for a relationship to physical activity [16]. A study in Seattle found that 49% of participants had greater exposure to supermarkets outside their home neighbourhood [17]. Similar results were found in Minnesota, where the participants had more than twice the exposure at work than at home [11].

That defining neighbourhoods presents challenges seems evident, and several studies appear to agree on several suggested challenges [9,15,18,19,20]. Ball *et al.* [1] explain that (1) people live and function in multiple contexts and settings; (2) people live and work in multiple geographic areas; and (3) different types of environmental influences exist, including built, natural, social, cultural and policy environments. Consequently, methods used for defining neighbourhoods must comply with individual behavioural characteristics. Focus on the individual is conceptualised by Rainham *et al.* through the change from a place-based to a people-based perspective with individual-based measures [21].

Previous studies reveal numerous examples that contradict the people-based approach through application of administrative divisions as the spatial extent for a neighbourhood [18,22]. Census tracts [23,24,25,26], zip codes [22] or parishes are used as a spatial representation of a neighbourhood for analysis of exposure to the food environment.

Neighbourhoods based on buffers also rely strongly on the location of the home but also offer an individual measurement. However, the difference is small for people living close to one another. The buffer method is widely used [16] to create neighbourhood definitions for residences [5,9,24,27], schools [13,28,29,30,31,32,33] and work locations [9]. The buffer distances and methods varies between fixed distances or a travel time constraint and either Euclidian or network distances [27].

Administrative divisions and buffers applied to the residential location adhere to a conceptual and analytic platform, where place is the central element in studying human behaviour. From the place-based perspective, all behaviour is located and centralised around the home. The importance of people’s closeness and sense of belonging to a certain community and place is challenged by today’s society. No matter what one believes, human mobility has increased substantially in the last century, and connectivity now makes activities and places more dynamic.

The problem is that each individual is unique and consequently must be assumed to have their own concept of neighbourhood. Complexity and heterogeneity of human mobility no longer appear to correspond to the use of residential neighbourhoods. Exposure to the food environment occurs in multiple environments, but to measure the impact of people’s individual exposure in multiple environments is challenging.

Technologies for tracking individuals’ behaviour have been available for more than a decade. However, development of lightweight, low-cost and accurate Global Position System (GPS) devices and assisted GPS in smartphones has boosted the use of tracking within behavioural nutrition research. GPS provides an individual measurement of space-time information about people’s behaviour. The outcome of GPS tracking can potentially consist of millions of data entries, which must be handled and conceptualised to resemble a neighbourhood. Common methods for simplifying neighbourhoods (or activity spaces) from GPS data are standard deviational ellipses (SD ellipses) and home range (minimum convex polygon) [21,34]. The derived activity spaces are individual and not dependent on a fixed location. Commuting routes and leisure time activities are therefore also included.

Although many studies utilise neighbourhood as a concept, few studies explore how neighbourhoods are defined or which definition is most suitable for the study. A variety of neighbourhood definitions are applied in relation to measuring the impact of the food environment.

Therefore, the aims of this study are (1) to compare different definitions of neighbourhoods for analysis of exposure to healthy/unhealthy food options, where supermarket exposure is perceived to be healthy and fast food exposure to be unhealthy; (2) to investigate the differences in neighbourhood area size and in the number of food outlets by type within neighbourhoods; and (3) to discuss the influence of the neighbourhood definition on the measure of exposure.

## 2. Methods

### 2.1. Study Area and Sample

**Figure 1:**
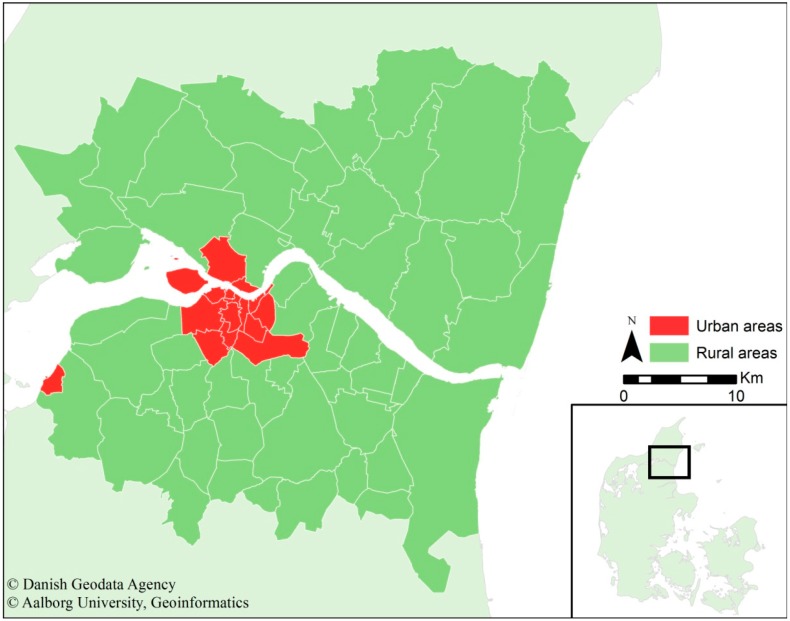
Presentation of the study area, the relative location in Denmark and the division between urban and rural areas.

The study area consists of 65 parishes (15 urban and 50 rural) in Northern Jutland (Denmark) centralised around Aalborg as the largest city in the region. The population in the study area is approximately 230,000, and of that number, approximately 120,000 live in Aalborg. The study area is approximately 1552 km^2^, of which Aalborg, with its high-density housing (mean ≈ 1700 people/km^2^) only comprises 68.3 km^2^ (≈4.4%). The remaining areas consists of small villages with populations up to 7000 and low-density housing (mean ≈ 85 people/km^2^). The study area’s spatial extent, relative location in Denmark and the divide in urban and rural areas are presented in Figure 1. Northern Jutland consists of 11 municipalities, five of which are defined as peripheral regions. Peripheral regions are characterised by, among other factors, a lower average income than the national average, a lower amount of commuting traffic and low or negative population growth. However, Aalborg attracts many young people and is the economic centre of the region. In Northern Jutland, approximately 50% of all people aged 16 to 25 lives in Aalborg, whereas these people are only approximately 17% of the entire population.

The study involves a random sample of 223 people selected from a population of 7277 people enrolled in school in Aalborg. Respondents were distributed between six school locations. The sample has a higher proportion of female (57%) than male (43%) participants. The participants’ ages range from 16 to 23 years old, with an average age of 17.7 years. Each person was tracked by the Global Positioning System (GPS) for one week of their typical school schedule. The GPS devices used in this study are the Lommy Phoenix and are approximately the same size as a mobile phone. The participants were asked to carry the device at all possible times during the week. All subjects provided their informed consent for inclusion before they participated in the study and could opt out at any time by turning off the GPS device. The tracking resulted in 8.22 million records for the 223 participants. The number of loggings registered for each person varied from 579 to 128,679, with an average of 36,523.

A threshold of 30 h (equal to waking hours for two days) of tracking was set as a minimum for the participants to be included in the study. The final sample consists of 187 people (36 were excluded). The final sample population includes 110 women (58.8%) and 77 men (41.2%) from 16 to 23 years old (the mean age is 17.3 years old). The final sample includes 93 people who live in a rural area and 94 people who live in an urban area.

### 2.2. GPS Data Preparation

GPS tracking is subject to several technical limitations when measuring space-time data [19,35]. Connection to an adequate amount of satellites is critical because lack of such a connection can result in inaccurate position data or complete loss of data for a period. The errors can be categorised as (1) outliers, either in attribute values for number of satellites, horizontal delusion of precision (HDOP) and time to fix (TTF), or extreme positions (e.g., on equator); or (2) scatter, in the form of unnatural linear point patterns [35]. The unnatural linear point patterns are detected by little or no change in the direction between three or more subsequent loggings, and the location of these loggings are outside a 50 m buffer on the road network. Detection of outliers and scatter found 341,741 loggings that were perceived as erroneous data.

The GPS devices were set to register the location at 7 s intervals, which was the lowest interval possible for the devices used. However, due to external conditions (*i.e.*, visibility to satellites and time to establish a fix), the logging interval varies up to 60 s. Calculation of several neighbourhood definitions assumes an even time interval between loggings (e.g., SD ellipses) because they are based on statistical assumptions. Spatial linear interpolation between subsequent loggings was applied to create an even time interval of 1 s between each logging. However, a 60 s threshold is set because the GPS creates a duplicate of the previous logging if it cannot obtain three consecutive measurements with a HDOP less than 30 in 60 s. The consequence can be large time leaps, for which it is difficult to estimate or guess the location. The interpolation results in a data set consisting of 60.18 million loggings, which corresponds to an average of three days and 17.4 h of active tracking for each participant.

### 2.3. Neighbourhood Definitions

#### 2.3.1. Administrative Divisions

Division of the land into smaller areas is used administratively on several levels in most countries, and previous studies refer to census tracts and zip codes used for spatial analysis. The purposes of the administrative division vary, but none were created for research purposes. The consequence of using administrative divisions as measures of exposure to the food environment implies that all individuals within these divisions will be exposed solely to the food outlets within those boundaries. Thus, it relies on people to have a strong residential connection.

This study uses parishes because they are the smallest official administrative division in Denmark. The area size of parishes within the study varies from 0.65 to 110.49 km^2^ (mean = 23.85 km^2^), the population ranges from 98 to 12,544 people and the population density varies from 14.39 to 9097 people/km^2^. People were assigned to the parish in which their residence is located.

#### 2.3.2. Buffers

Buffers are used to create a circular area at a specified distance, and they are quick to calculate, easy to understand and easy to compare because the area size is equal for all study subjects. Simple buffers are based on Euclidian distances, whereas buffers that are more complex are based on network analysis. The buffer distance should be appropriate for examining nutrition-related behaviours for the target group involved. Little agreement exists on the appropriate distance, and multiple distances are applied in research [16]. This study applies two distances for defining the buffer size. A distance of 800 m was selected because it is approximately equal to a 10 min walk (5 km/h). Second, a distance of 1600 m (≈1 mile) was selected because it is frequently used in other studies [5,9,13,16,24,28,29,32]. A study of adults in England demonstrated that more than 95% of usual walking destinations were within 1600 m of the home [36]. This study calculates buffers on the home and school addresses. A third neighbourhood definition is defined by combining the buffers for home and school.

#### 2.3.3. Convex Hull (Minimum Bounding Geometry)

The convex hull area is created to represent the minimum bounding geometry enclosing all the GPS loggings for each individual. The convex hull represents the maximum area in which the individuals engaged in activities.

#### 2.3.4. Standard Deviational Ellipses

The standard deviational (SD) ellipses are created by calculating the standard deviation in the x-coordinates and y-coordinates from the mean centre of the coordinates. The ellipses do not represent the maximum area in which the individual could engage in activities but rather the area in which the individual is likely to be regularly involved in activities. This study applies one and two SD ellipses, which implies that approximately 68% and 95% or more of the GPS loggings are positioned within the one or two SDs, respectively. The position of each GPS logging is a weight in calculating the ellipses extent. The GPS loggings therefore must represent an individual’s whereabouts, which is performed through interpolation on the space-time data.

#### 2.3.5. Path Area

The GPS loggings are used to create the path area represents the participants’ travel patterns. For each GPS logging, the nearest road or path segment was determined through a near analysis. On the road and path segments, a 50 m buffer was applied. The buffer is needed to capture the exposure to food outlets, for which spatial location often has an offset of 5–30 m from roads.

**Figure 2:**
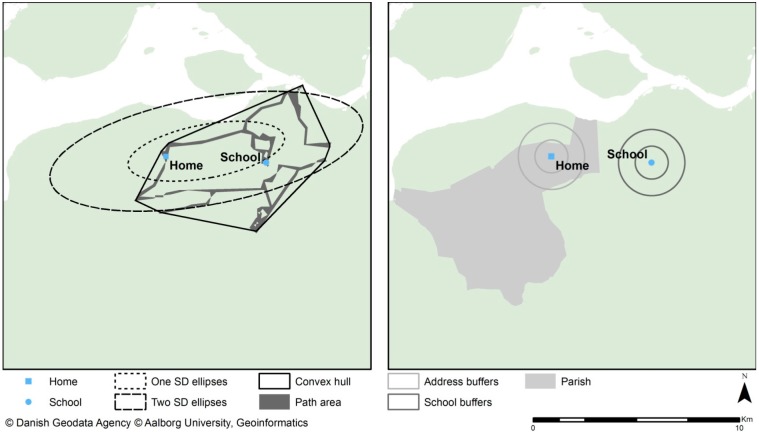
Visual representation of neighbourhood spatial extent and definition.

Figure 2 presents a spatial comparison of the neighbourhood definitions.

### 2.4. Food Outlet Data

**Figure 3:**
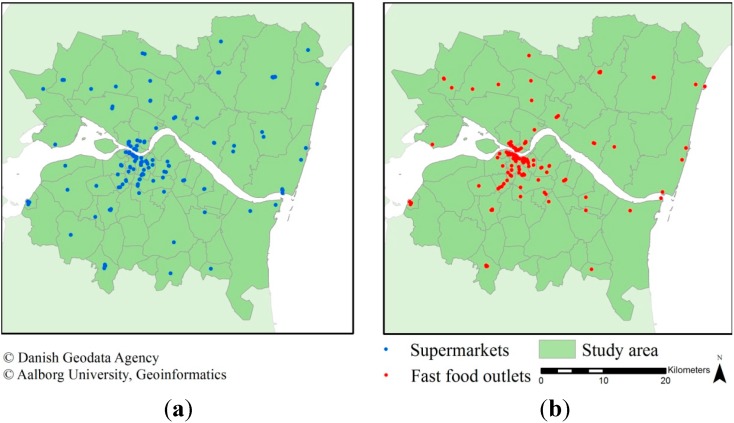
The spatial distribution of (**a**) supermarkets; and (**b**) fast food outlets within the study area.

Data on fast food outlets and supermarkets were retrieved from the national business register (CVR) and the national food safety and hygiene regulation register (Smiley). The spatial and semantic validity has been described in previous research [37]. A pre-classification method of the business type based on the outlets name was applied as described in [37]. This resulted in 144 supermarkets (including discount) and 154 fast food outlets in the study area. The addresses in CVR were geocoded based on address reference data in the Universal Transverse Mercator (UTM) projection obtained from the Danish Geodata Agency. The Smiley register contains World Geodetic System 84 (WGS84) coordinates for approximately 95% of entries, which were transformed into UTM and used as their locations. The remaining records are geocoded by the address using reference data from the Danish Geodata Agency. The distribution of the supermarkets and fast food outlets is depicted in Figure 3.

### 2.5. Statistical Analysis

This study compares the mean values for food outlets exposure in each neighbourhood to analyse differences. Consequently, the null hypothesis is that any difference between the groups is a result of sampling error, and the actual differences between the means are effectively zero. The Welch two-sample *t*-test is applied to compare two groups, and the one-way ANOVA (*F*-test) is applied for comparing three or more groups.

One-way ANOVA assumes that the data are sampled from populations that follow a Gaussian distribution. Although this assumption is not very important with large samples, it is important with small sample sizes and particularly with unequal sample sizes. One-way ANOVA assumes that all the groups have the same standard deviation. This assumption is not very important when all the groups have the same or almost the same number of individuals. The sample sizes in this study are equal for all one-way ANOVA tests.

The one-way ANOVA compares several groups but does not inform about groups having significantly different means. The differences between groups might be due to errors in the sampling whereas others might not be. Therefore, a post hoc comparison test is conducted to examine the differences between pairs of each of the neighbourhood types. This identifies pairs of neighbourhoods that have significantly large differences, which are not the result of sampling errors. This is calculated using Tukey’s HSD (honest significant difference) test. Tukey’s HSD test is weak, meaning it is less likely to detect significant results. The test assumes normality for each group of data, the observations are independent within and among groups and there is homogeneity of variance. The test is quite robust to violations of normality and to some extent violations of homogeneity of variance for large samples. Tukey’s HSD test requires previous calculation of one-way ANOVA and is calculated using Equation (1). *M*_1_ and *M*_2_ are the means of the neighbourhood groups, *MS_w_* is the mean square within groups from the one-way ANOVA and *n* is the number per group.

(1) $$HSD=\;\frac{M_{1}-M_{2}}{\sqrt{MS_{w}\left(\frac{1}{n}\right)}}$$

The Welch *t*-test is used to test the hypothesis that two independent or unpaired groups of data have equal means. The test is an adaption of the students’ *t*-test, but it is used when the variance possibly is unequal. The test compares urban and rural samples, which are non-overlapping. The test assumes the data are independent. The Welch *t*-test is calculated using Equation (2), where ${\bar{X}}_{i}$ is the group means, *S_i_* is the group variance and *N_i_* is the group sample size.

(2) $$t=\;\frac{{\bar{X}}_{1}-{\bar{X}}_{2}}{\sqrt{\frac{s_{1}^{2}}{N_{1}}+\frac{s_{2}^{2}}{N_{2}}}}$$

All statistical analyses are calculated using R [38].

## 3. Results

### 3.1. Comparison of Neighbourhood Area Sizes

There are 11 different definitions of neighbourhoods in this study with different spatial characteristics and extents as illustrated in Figure 2. Descriptive statistics for neighbourhood area sizes are presented in Table 1. The mean areas vary from 2.01 to 51.39 km^2^. Significant dispersions occur for the neighbourhood type’s parish, convex hull, one SD ellipses and two SD ellipses, which is reduced slightly by dividing the sample into urban and rural areas. No variance exists between buffers around schools or addresses due to equality of area sizes for all participants.

**Table 1:** Mean area and standard deviation for neighbourhoods for total sample (*n* = 187), urban (*n* = 94) and rural (*n* = 93) areas. Lower portion of table presents results of ANOVA for neighbourhoods.

Neighbourhood		Area (km^2^)		Urban Area (km^2^)		Rural Area (km^2^)	
		Mean	σ	Mean	σ	Mean	σ
Place-based neighbourhoods							
Parish		17.80	20.05	5.70	3.94	30.02	22.28
Address 800 m buffer		2.01	-	2.01	-	2.01	-
Address 1600 m buffer		8.04	-	8.04	-	8.04	-
School 800 m buffer		2.01	-	2.01	-	2.01	-
School 1600 m buffer		8.04	-	8.04	-	8.04	-
Combined 800 m buffer		3.91	0.34	3.80	0.46	4.02	0
Combined 1600 m buffer		15.11	1.98	13.99	2.29	16.06	0.32
Person-based neighbourhoods							
Convex hull		51.13	82.30	21.14	34.93	81.45	103.02
One SD ellipses		17.78	40.55	4.53	6.26	31.17	54.08
Two SD ellipses		51.39	89.99	16.69	21.48	86.46	115.90
Path area		4.76	2.96	3.45	2.40	6.08	2.91
ANOVA	*F*-test values	39.83		24.34		35.48	
	Significance level	<0.001		<0.001		<0.001	
Tukey’s HSD test		26 of 55 pairs have significant different means		27 of 55 pairs have significant different means		26 of 55 pairs have significant different means	

Table 1 suggests that the areas of rural neighbourhoods are noticeably larger than those in urban neighbourhoods. The Welch *t*-test compares the area sizes for urban and rural neighbourhoods, and the results are presented in Table 2. All *t*-values from the test are positive, indicating that the rural areas are larger than the urban areas. The *t*-values range from 4.671 to 10.369, and the significance levels for all neighbourhoods are below 0.001. The significance levels indicate that the null hypothesis of no difference in area sizes is rejected. Hence, the differences between urban and rural area sizes are most likely not due to sampling error.

Results of the one-way ANOVA are presented in Table 1. *F*-test values in the interval from 24.34 to 39.83 and significance levels below 0.001 indicates that the differences between mean area sizes of the 11 neighbourhoods has almost no chance of being caused by sampling error. The null hypothesis of no difference between mean area sizes is rejected. The results of Tukey’s HSD test presented in the Supplementary Material provide information about which pairs of neighbourhood area sizes have significantly different means.

**Table 2:** The results of the Welch *t*-test comparing urban and rural neighbourhood area sizes. Buffer around school and address is omitted due to no difference.

Neighbourhood	t	df	Sig. (Two-Sided)	95% Conf. Interval of the Differences	
				Lower	Upper
Place-based neighbourhoods					
Parish	10.369	97.691	** <0.001	19.667	28.977
Address & school buffer 800 m	4.671	93.000	** <0.001	0.126	0.313
Address & school buffer 1600 m	9.427	96.655	** <0.001	1.773	2.718
Person-based neighbourhoods					
Convex hull	5.349	112.674	** <0.001	37.973	82.646
1 standard deviational ellipses	4.721	94.439	** <0.001	15.439	37.853
2 standard deviational ellipses	5.709	98.246	** <0.001	45.521	94.022
Path area	6.731	177.809	** <0.001	1.856	3.395

Note: ** Statistically significant below the 0.01 level.

Table 3 illustrates the average overlaps between neighbourhood types presented. Because the neighbourhoods vary in extent and location, the overlap between two neighbourhoods is not equal. The path area is the tightest measure of the participants’ behaviour. Comparison of the overlaps between path area and the other 10 neighbourhoods reveals overlaps from 11.9% to 27.2%, which indicate that the other 10 neighbourhoods are only partially used.

### 3.2. Comparison of Neighbourhoods’ Ability to Capture measured GPS Activity

Each participant’s activity was measured using GPS and the neighbourhood types’ convex hull and path area by definition captured 100% of the activity. The mean amount of loggings within each neighbourhood type is presented in Table 4. The neighbourhood types, which most poorly captured the GPS-measured activities, were the 800 and 1600 m buffers around schools. The remaining mean values range from 72.93% to 94.35% GPS loggings within the neighbourhoods.

Table 4 presents the results of the one-way ANOVA. The large *F*-test value of 509.8 and a significance level of <0.001 denote that the neighbourhood’s ability to capture the measured GPS activity has almost no chance of demonstrating equal means for all 11 neighbourhoods. The null hypothesis of no difference between each neighbourhood’s ability to capture human activity is rejected. Tukey’s HSD test was calculated to compare the individual pairs and 47 out of 55 pairs were significantly different in mean amount for loggings located within the neighbourhood boundaries. The results of Tukey’s HSD test are available in the Supplementary Material.

Tests were conducted by dividing the data into urban and rural areas. The Welch *t*-test reported significant differences for the school 1600 m buffer (*t* = −3.220, sig = 0.001) and combined 800 m buffer (*t* = −4.894, sig < 0.001). In both cases, the urban neighbourhoods captured a significantly larger proportion than the rural sample.

**Table 3:** Percentage of the column neighbourhood type that the row neighbourhood type overlaps, on average.

Neighbourhood	Address Buffer 800 m	Address Buffer 1 mile	Convex Hull	Address & School Buffer 800 m	Address & School Buffer 1 Mile	Path Area	School Buffer 800 m	School Buffer 1 Mile	1 Standard Deviational Ellipses	2 Standard Deviational Ellipses	Parish
Address 800 m buffer	-	24.7%	9.7%	52.0%	13.6%	15.5%	5.5%	3.7%	35.8%	17.8%	23.8%
Address 1600 m buffer	100.0%	-	23.9%	57.5%	55.1%	29.4%	18.2%	14.3%	58.3%	39.0%	52.5%
Convex hull	60.4%	45.4%	-	63.8%	48.2%	95.9%	67.9%	53.4%	78.2%	55.1%	38.9%
Combined 800 m buffer	100.0%	27.1%	18.1%	-	26.1%	31.1%	100.0%	27.1%	40.0%	23.8%	25.3%
Combined 1600 m buffer	100.0%	100.0%	40.4%	100.0%	-	49.7%	100.0%	100.0%	64.5%	49.1%	54.7%
Path area	24.3%	11.9%	21.2%	25.3%	12.1%	-	27.2%	13.4%	25.0%	15.0%	12.6%
School 800 m buffer	5.5%	4.5%	10.5%	52.0%	13.6%	16.5%	-	24.7%	8.8%	8.4%	5.7%
School 1600 m buffer	15.2%	14.3%	26.5%	57.5%	55.1%	31.1%	100.0%	-	23.2%	21.9%	14.4%
One SD ellipses	65.1%	40.3%	22.2%	40.6%	26.0%	27.3%	16.9%	13.6%	-	26.2%	30.7%
Two SD ellipses	87.7%	69.6%	52.3%	71.1%	55.9%	57.0%	54.9%	44.2%	97.8%	-	55.2%
Parish	79.0%	60.5%	18.4%	44.1%	33.5%	23.3%	9.6%	8.2%	48.5%	31.7%	-

**Table 4:** Mean count of GPS loggings located within each neighbourhood (*n* = 187). Bottom of table holds results of ANOVA for logging count in neighbourhoods.

Neighbourhood		GPS Logging Count in Neighbourhoods	
		Mean	σ
Place-based neighbourhoods			
Parish		250,302.4 (73.98%)	142,687.9
Address 800 m buffer		248,100.1 (72.93%)	143,025.2
Address 1600 m buffer		256,252.2 (76.30%)	142,999.4
School 800 m buffer		46,299.8 (17.09%)	60,025.4
School 1600 m buffer		70,197.3 (25.50%)	91,348.3
Combined 800 m buffer		281,020.8 (84.71%)	149,402.6
Combined 1600 m buffer		290,858.0 (88.82%)	148,838.4
Person-based neighbourhoods			
Convex hull		321,796.1 (100%)	152,318.5
One SD ellipses		264,880.8 (81.64%)	132,870.1
Two SD ellipses		302,087.2 (94.35%)	145,004.9
Path area		321,796.1 (100%)	152,318.5
ANOVA	*F*-test values	509.8	
	Significance level	<0.001	
Tukey’s HSD test		47 of 55 pairs have significant different means	

### 3.3. Comparison of Exposure to Supermarkets in Neighbourhoods

The number of supermarkets located within each neighbourhood served as a measure of the exposure to supermarkets, and the results are presented in Table 5. The mean amount of supermarkets located in the neighbourhoods varies from 2.18 for the address 800 m buffer to 26.44 for convex hull. The mean amount of supermarkets in each neighbourhood type has a strong positive linear relationship with the size of the neighbourhood areas (cor. coef. = 0.80 and *p* = 0.003). When taking the neighbourhood area sizes into account, the neighbourhoods’ school 800 m buffer and path area distinguish themselves by having significantly more supermarkets per square kilometre.

Table 5 presents the results of the one-way ANOVA. The high *F*-values and significance levels below 0.001 for all denote that almost no chance exists that the exposure to supermarkets are equal for all 11 neighbourhoods. The null hypothesis of no difference between supermarket exposures in neighbourhoods is rejected. Tukey’s HSD tests were calculated to compare the individual pairs and the proportions of significant pairs are presented in the last row of Table 5. A distinction is made between the amount of significant pairs for the urban and rural samples for both the raw data count and supermarkets per square kilometre. The complete results of Tukey’s HSD test are available in the Supplementary Material.

The one-way ANOVA and Tukey’s HSD test highlighted the differences between urban and rural neighbourhoods. The results of the Welch *t*-test presented in Table 6 accentuate the significant difference for supermarket exposure in the urban and rural samples. Non-significant differences exist between one SD ellipses and both school buffers that are most likely the result of the schools being identical for urban and rural participants. All the place-based neighbourhoods have negative *t*-values, which indicate higher supermarket exposure in the urban sample. However, the *t*-values are positive for the individual-based neighbourhood types, which indicate a higher supermarket exposure in the rural sample.

**Table 5:** Mean exposure to supermarkets in each neighbourhood for total (*n* = 187), urban (*n* = 94), rural (*n* = 93) per km^2^ (*n* = 187), per km^2^ urban (*n* = 94) and per km^2^ rural (*n* = 93) samples. The lower portion of the table presents results of ANOVA and Tukey’s HSD for neighbourhoods.

		Supermarkets		Supermarkets Urban Areas		Supermarkets Rural Areas		Supermarkets pr. km^2^		Supermarkets pr. km^2^ (Urban)		Supermarkets pr. km^2^ (Rural)	
Neighbourhood		Mean	σ	Mean	σ	Mean	σ	Mean	σ	Mean	σ	Mean	σ
Place-based neighbourhoods													
Parish		3.43	2.31	4.64	2.19	2.20	1.70	0.79	1.13	1.47	1.26	0.09	0.08
Address 800 m buffer		2.18	2.51	3.50	2.83	0.85	1.05	1.09	1.25	1.74	1.40	0.42	0.52
Address 1600 m buffer		6.01	5.87	10.14	5.55	1.83	1.87	0.74	0.72	1.24	0.68	0.22	0.23
School 800 m buffer		4.93	2.87	4.76	3.19	5.11	2.51	2.45	1.43	2.36	1.58	2.54	1..25
School 1600 m buffer		12.65	6.28	12.29	6.50	13.01	6.07	1.55	0.77	1.51	0.79	1.59	0.74
Combined 800 m buffer		6.79	3.75	7.61	4.38	5.96	2.75	1.75	1.00	2.02	1.17	1.48	0.68
Combined 1600 m buffer		16.70	7.61	18.61	8.36	14.75	6.23	1.12	0.55	1.33	0.59	0.90	0.38
Person-based neighbourhoods													
Convex hull		26.44	15.97	22.22	15.69	30.70	15.17	1.32	1.16	1.90	1.27	0.73	0.62
One SD ellipses		6.36	8.32	5.88	7.00	6.85	9.49	1.18	1.84	1.90	2.29	0.44	0.67
Two SD ellipses		20.04	18.75	16.15	14.07	23.97	21.89	0.97	1.05	1.47	1.22	0.44	0.41
Path area		11.44	6.25	10.41	6.30	12.47	6.06	2.82	1.45	3.39	1.53	2.23	1.09
ANOVA	*F*-test values	137.8		55.35		100.6		60.49		19.51		134.3	
	Sig. level	<0.001		<0.001		<0.001		<0.001		<0.001		<0.001	
Tukey’s HSD test		44 of 55 pairs have significant different means		39 of 55 pairs have significant different means		42 of 55 pairs have significant different means		33 of 55 pairs have significant different means		19 of 55 pairs have significant different means		41 of 55 pairs have significant different means	

Comparing supermarket exposure per square kilometre in urban and rural neighbourhoods resulted in significant differences for all neighbourhood types except for the two school buffers as indicated in the previous *t*-test in Table 6. The remaining *t*-values are all negative indicating a higher supermarket exposure per square kilometre in the urban sample.

**Table 6:** The results of the Welch *t*-test comparing urban and rural neighbourhood exposure to supermarkets.

Neighbourhood	*t*	df	Sig. (Two-Sided)	95% conf. Interval of the Differences	
				Lower	Upper
Place-based neighbourhoods					
Parish	−8.489	175.316	** <0.001	−3.000	−1.868
Address 800 m buffer	−8.512	118.519	** <0.001	−3.267	−2.034
Address 1600 m buffer	−13.749	114.016	** <0.001	−9.508	−7.113
School 800 m buffer	0.839	176.145	0.403	−0.476	1.181
School 1600 m buffer	0.787	184.374	0.432	−1.091	2.538
Combined 800 m buffer	−3.086	156.853	** 0.002	−2.705	−0.594
Combined 1600 m buffer	−3.567	171.904	** <0.001	−5.970	−1.716
Person-based neighbourhoods					
Convex hull	3.756	184.902	** <0.001	4.023	12.928
One SD ellipses	0.792	169.149	0.430	−1.443	3.376
Two SD ellipses	2.902	156.711	** 0.004	2.497	13.141
Path area	2.277	184.838	* 0.024	0.275	3.842

Notes: * statistically significant below the 0.05 level, ** Statistically significant below the 0.01 level.

### 3.4. Comparison of Exposure to Fast Food Outlets in Neighbourhoods

Table 7 presents the results of fast food exposure in neighbourhoods. The mean amount of fast food outlets that are located within each neighbourhood vary from 3.81 for the place-based neighbourhoods to 46.92 fast food outlets for the person-based neighbourhoods. More fast food outlets per square kilometre are located near the schools than in other locations.

**Table 7:** Mean exposure to fast food outlets in each neighbourhood for total (*n* = 187), urban (*n* = 94), rural (*n* = 93) per km^2^ (*n* = 187), per km^2^ urban (*n* = 94) and per km^2^ rural (*n* = 93) samples. The lower portion of the table presents results of ANOVA and Tukey’s HSD for neighbourhoods.

		Fast Food Outlets		Fast Food Outlets Urban Areas		Fast Food Outlets Rural Areas		Fast Food Outlets pr. km^2^		Fast Food Outlets pr. km^2^ (Urban)		Fast Food Outlets pr. km^2^ (Rural)	
Neighbourhood		Mean	σ	Mean	σ	Mean	σ	Mean	σ	Mean	σ	Mean	σ
Place-based neighbourhoods													
Parish		4.06	4.16	6.44	4.56	1.67	1.54	1.86	4.76	3.603	6.260	0.091	0.105
Address 800 m buffer		3.81	6.51	6.85	8.06	0.74	1.05	1.90	3.24	3.407	4.007	0.369	0.523
Address 1600 m buffer		9.79	13.71	17.81	15.45	1.68	2.54	1.20	1.68	2.188	1.898	0.206	0.311
School 800 m buffer		13.71	11.99	13.27	12.36	14.15	11.65	6.82	5.96	6.597	6.146	7.037	5.799
School 1600 m buffer		26.99	21.41	26.06	21.94	27.92	20.93	3.32	2.63	3.203	2.696	3.431	2.572
Combined 800 m buffer		16.47	12.97	18.03	14.06	14.89	11.64	4.28	3.47	4.842	3.901	3.703	2.893
Combined 1600 m buffer		33.07	22.27	36.54	23.02	29.57	21.02	2.24	1.59	2.649	1.753	1.820	1.291
Person-based neighbourhoods													
Convex hull		46.92	25.11	42.44	25.08	51.45	24.44	2.87	3.60	4.399	4.416	1.329	1.323
One SD ellipses		11.30	16.97	11.74	15.75	10.86	18.20	2.23	3.84	3.687	4.770	0.751	1.573
Two SD ellipses		34.98	31.61	31.10	26.11	38.91	36.05	2.12	4.10	3.499	5.375	0.729	0.883
Path area		24.29	12.65	23.60	13.18	24.99	12.12	6.45	4.53	8.213	5.198	4.658	2.797
ANOVA	*F*-test values	110.7		42.69		78.45		46.06		14.78		80.82	
	Sig. level	<0.001		<0.001		<0.001		<0.001		<0.001		<0.001	
Tukey’s HSD test		45 of 55 pairs have significant different means		35 of 55 pairs have significant different means		47 of 55 pairs have significant different means		29 of 55 pairs have significant different means		20 of 55 pairs have significant different means		36 of 55 pairs have significant different means	

The results of the one-way ANOVA for fast food exposure are presented in Table 7. The *F*-values varies from 14.78 to 110.7 and is a hint of how many pairs of neighbourhoods have significantly different mean fast food exposure. Significance levels are below 0.001 for all analysis of variance, indicating that almost no chance exists that the exposure to fast food outlets are equal for all 11 neighbourhoods in any of the six analyses of variance. Tukey’s HSD tests were calculated to compare the individual pairs and the proportions of significant pairs are presented in the last row of Table 7. Fewer significantly different pairs of neighbourhoods are found to experience fast food exposure in urban areas than in the rural sample. The complete results of Tukey’s HSD test are available in the Supplementary Material.

The results of Tukey’s HSD test were significantly different between the fast food outlet exposure in rural neighbourhoods (47/36 of 55) and some of the urban neighbourhoods (35/20 of 55). The Welch *t*-test compares the fast food exposure in the urban and rural neighbourhoods. The results of the *t*-tests are presented in Table 8. Significant differences exist between the mean exposures to fast food outlets for the home-based neighbourhood’s parish, address 800 and 1600 m buffer. For all three, the *t*-values are negative denoting a higher exposure in the urban sample.

**Table 8:** The results of the Welch *t*-test for comparing fast food outlet exposure in urban and rural neighbourhoods.

Neighbourhood	*t*	df	Sig. (Two-Sided)	95% Conf. Interval of the Differences	
				Lower	Upper
Place-based neighbourhoods					
Parish	−9.598	114.176	** <0.001	−5.754	−3.785
Address 800 m buffer	−7.288	96.203	** <0.001	−7.772	4.445
Address 1600 m buffer	−9.988	98.067	** <0.001	−19.336	−12.926
School 800 m buffer	0.503	184.587	0.615	−2.581	4.350
School 1600 m buffer	0.593	184.756	0.554	−4.326	8.048
Combined 800 m buffer	−1.664	179.407	0.097	−6.862	0.583
Combined 1600 m buffer	−2.163	183.83	* 0.032	−13.332	−0.613
Person-based neighbourhoods					
Convex hull	2.489	184.958	* 0.014	1.871	16.160
One SD ellipses	−0.355	180.686	0.723	−5.798	4.029
Two SD ellipses	1.697	167.599	0.092	−1.277	16.913
Path area	0.753	184.021	0.452	−2.258	5.045

Notes: * Statistically significant below the 0.05 level, ** statistically significant below the 0.01 level.

The results of the Welch *t*-test for comparing fast food exposure per square kilometre in urban and rural neighbourhoods resulted in significant differences for all neighbourhoods except both school buffers. The *t*-values are all negative, which indicate a higher fast food exposure per square kilometre in the urban sample.

## 4. Discussion

### 4.1. Place Based vs. People Based Neighbourhood Definitions

The understanding of place as a concept stretches from the individual adhering to their own unique place determined by their everyday life and behaviour to the claim that the individual unconsciously relates their behaviour and choices to more structured patterns based on social and physical environment characteristics [2]. However, often the discussion about place is ignored due to pragmatic considerations, such as data only being accessible in administrative units. Administrative divisions as the concept for place are therefore often the natural choice for many researchers without considering the administrative divisions’ ability to encapsulate the relevant behaviour. The consequence is a wrong assumption or generalisation that all individuals have equal behaviour patterns, limiting the exposure to a confined area and limiting diversity in food supply choices.

This study reveals that the administrative divisions are not a suitable neighbourhood type to capture the measured behaviour. This finding is supported by the fact that only 12.8% (24 of 187) of the participants attend school in their residential parish, and the exposures to supermarkets and fast food outlets around the schools are more than three and six times higher, respectively, than in the parishes. This fact coincides with previous studies that found similar relationships between exposures near home and school [11,15,17]. However, the differences between home and school neighbourhoods are significantly more distinctive for participants living in a rural area and attending schools in urban areas.

The place-based neighbourhood definitions do not take into account the diversity in individual behaviour. This problem is most likely the result of assuming people carry out most of their activities in their residential location, which is contradicted by the high mobility in the participant sample. The participants in this study are young adults, and most have a high mobility level even without the ability to drive a car. The participant’s mobility must be taken into account because it weakens the influence of residential neighbourhoods. However, other studies with low mobility group samples, such as the elderly and the disadvantaged people, are probably more sensitive to the residential neighbourhood exposure [20].

The use of the term neighbourhood in food environment research adheres to spaces defined by fixed boundaries, such as administrative units, or a fixed distance, such as buffers, that define a school or residential neighbourhood [4]. When referring to individual-measured areas, a more appropriate term instead of neighbourhood is “activity spaces” as suggested by Zenk and colleagues [39]. This division between terms can potentially improve researchers’ understanding of the differences between the place-based and person-based exposure measures.

Defining individual activity spaces is advantageous for providing increased specificity in a multiple space exposure measurement. However, as Ball and colleagues note, the collection of activity space attribute data can be time and labour intensive because the individual activity spaces do not align spatially with existing administrative divisions [1]. The activity spaces defined by the individual’s behaviour most likely vary in area size, which increases the complexity of analysis when comparing different individuals’ exposure. Moreover, comparisons across different studies are very difficult if the activity spaces vary in area size. The equal size of neighbourhoods based on buffers makes them easier to compare between studies in different countries. However, the buffers are limited to a few locations, and as this study reveals, the buffers and the administrative divisions have similar problems in capturing exposure during commuting or leisure time activities. The researcher’s perception is that the use of multiple-location buffers provides a much better basis for measuring exposure than single-area buffers and administrative divisions. Applying buffers on either home or school only provides one piece in the complex puzzle of measuring the complete exposure. Many studies have limited the research area to a residential/school neighbourhood (for example, a 1 km buffer) [5,9,13,16,24,27,28,29,30,31,32,33] or administratively defined boundaries [22,23,24,25,26]. The studies thereby only consider data inside the sample area of interest. Data in adjacent areas are not implemented, which could be problematic because the effect of exposure across study boundaries is not considered. Another problem with the buffer areas created is how to define a relevant distance since found associations may vary depending on this definition [4]. To bypass these problems, researchers should consider measuring actual activity spaces, which is possible using GPS.

### 4.2. Implications for Research

The neighbourhoods’ ability to capture the activity measured by GPS varies, particularly for those neighbourhood types that are confined to one or two locations and enclose a smaller percentage of the measured activity. The parishes are typically more than eight times larger in area than the address 800 m buffer and two times the 1600 m buffer, but they enclose only 1% more and 2.5% less, respectively, of the measured activity. This finding indicates that most activity around the residential locations is tied very closely (within 800 m) to the home, whereas an enlargement of the residential neighbourhood to a 1600 m buffer or a parish has little effect on capturing more of the measured activity. Approximately 85% of the measured activities are near the home or school, but the final 15% poses a challenge for researchers to measure because it constitutes the behaviours that are most affected by individual preferences.

Individual characteristics as confounders are crucial to take into account personal preferences when analysing relationships between the food environment and health outcomes [1,2]. However, not all preferences can be adjusted through common confounders such as income, ethnicity and education level. Consequently, methods used for defining neighbourhoods must accommodate the individual behavioural characteristics [20]. However, to achieve this effect, researchers must carefully scrutinise the behaviour to be measured to fully understand the phenomenon. The way a space is defined should reflect the context in which it is applied [14]. Therefore, to measure the exposure to food environment, researchers must make qualified assumptions about where people shop, the distance they are willing to travel to shop and other individual preferences [2]. Thus, paying attention to the individual is important when developing studies of the interaction between the population and the environment. As Larson and Story concluded, most food environment studies have methodological problems that reduce the credibility of their findings [40]. Problems occur with assessing the physical access to food sources in the environment [4] and linking access to a food source with food purchases and intake. Further analysis of individual behaviour could potentially be used to link the food source exposure to individual food purchasing through analysing movement and stop flows in space-time data.

The results of this study are consistent with several other studies [1,2,15,20,21] advocating for more individual-based neighbourhood definitions taking into account multiple environments for exposure beyond home, school or work communities. Exposure during commuting time and leisure activities are particularly difficult to incorporate when the neighbourhoods are place based. Kwan further questions the use of arbitrary definitions of neighbourhoods instead of considering the actual spaces in which individuals’ exposure occur [41]. The main objections to the static and administrative bounded spatial definitions in ecological exposure measures found in this study and accentuated by Kwan are: (1) the assumption that the residential neighbourhoods are the most relevant in affecting food exposure; and (2) individuals who live in the same spatial areas experience the same level of exposure, regardless of time spent in the area and residential locations within the area [41]. The results from this study contradict the assumptions since individuals also spend a substantial time outside their residential neighbourhood, and the variance of individual activity space sizes illustrates the variety in individuals’ exposure.

Comparisons between urban and rural samples (*t*-tests) clearly reveal differences in exposure to supermarkets and fast food outlets in some neighbourhoods. Tukey’s HSD test similarly reveals that more neighbourhood types are significantly different in the rural sample than in the urban sample. Hence, a separation between urban and rural samples would create more homogenous samples. Individual activity spaces will vary depending on factors such as income, personal mobility (ability to drive, access to a vehicle, walking disabilities, *etc.*), age and other individual preferences. People living in rural areas are more likely to travel to a more populated area because these areas often provide greater access to work opportunities, food or cultural events, for example. On the other hand, urban residents are less likely to commute to rural areas, as their needs are mostly satisfied in the cities. The daily activity spaces of rural residents are presumably larger if they have no restrictions on their movement or travel abilities.

### 4.3. Limitations

The activity data measured by GPS clearly indicates that participants are using multiple locations and are thereby not restricted to their immediate residential environments. The survey period of one week is a short time frame for analysis of the participants’ behaviour. Short tracking periods could include locations, which might be visited infrequently and vice versa [20]. This phenomenon is the shortcoming of GPS technologies because recording consecutive involvement at such a level for longer periods is difficult. The development of tracking technologies is a fast growing field, and technologies such as Bluetooth, Wi-Fi and cellular phone networks could potentially be used to track participants in a way that requires less involvement from the individuals [42], mostly because all these technologies are included in most mobile phones today and therefore do not require participants to carry and maintain additional devices. The development of these technologies provides a promising improvement for empirical place research [21].

This study used GPS devices set to measure at seven-second intervals, which was the minimum interval available between loggings. A seven-second interval between registrations is a short time and discharges the battery faster than at a higher interval. A low interval between registrations is preferable for some uses, but the logging interval could probably be 15 s or more to measure the extent of the activity spaces. However, some problems occur with a high registration frequency. Activity measured by GPS can experience periods with loss of data that interferes with the registration interval. Activity space measures as standard deviational ellipses are calculated from the centre of gravity of the measured point locations and uneven intervals between registrations therefore affect the extent of the calculated spaces. Several methods have been proposed for resolving this issue by estimating missing data [39] or interpolation between registrations. Further, studies’ ability to measure individuals’ use of food retailers is dependent on a low interval between registrations. To detect stops at food retailers, several consecutive registrations at the same location are needed. Determining a maximum interval between registrations is difficult without further research, but a large interval between registrations results in a smaller dataset that is easier to analyse. Studies that apply GPS to measure activity must consider the accuracy required (interval between registrations) and the expected travel types and speed of participants.

The individual based neighbourhoods are better at capturing multiple space activity, but the measured exposure could be an exaggeration, which could be the case for the convex hull and two SD ellipses when compared to path area. The neighbourhood type convex hull has a large mean area size, particularly for the rural samples. Comparing convex hull with path area, which are both based on GPS tracking, reveals a 25% higher supermarket exposure for the convex hull neighbourhood type. However, if the area sizes for both neighbourhood types are used to adjust the exposure, then the exposure in path area is twice that of the convex hull. Path area is more focused on where the actual activity has occurred, but it does not capture deviant activities that would happen at other times than the single week when the activity was tracked. Therefore, whether the path area may underestimate the exposure remains unclear. To answer this question, researchers must delve into the understanding of people’s behaviour. Second, studying the relationship between measured exposure and the actual choices of food buying is relevant because this research could broaden the insight to defining a proper neighbourhood for measuring exposure to food outlets.

Any study of this type must use the appropriate spatial area to measure the exposure. However, many studies have applied place-based neighbourhoods with little focus on identifying these areas [41]. Among the most discussed methodological issues in research applying spatial data is the Modifiable Area Unit Problem (MAUP). MAUP refers to the issue that the areal units to which data are assigned might influence results. Neighbourhoods based on administrative divisions or buffers are highly susceptible to the MAUP. The place-based neighbourhoods allow little variation between individuals compared to the person-based neighbourhoods (Table 1). Large differences exist between individual activity spaces such as the convex hull and standard deviational ellipses where the standard deviation for each type of activity space is larger than the mean area size. This finding clearly indicates a large spread between individual activity spaces. Considering the actual spatial and temporal exposure would allow for a more accurate measure of exposure and address the MAUP [41]. This result would allow individuals to have individual exposure measures although they live in the same neighbourhood.

## 5. Conclusions

This study presents significant differences between the exposure to supermarkets and fast food outlets for different neighbourhood types. Second, significant differences were found for exposure to food outlets between urban and rural neighbourhoods.

Neighbourhoods are a fuzzy concept that varies in meaning depending on the conducted study and on each person’s individual perception of their neighbourhood. Complexity and heterogeneity of human mobility no longer appear to correspond to the use of residential neighbourhoods but stress the need for methods and measures of individual activity and exposure. Exposure to the food environment occurs in multiple environments, but measuring individuals’ activity spaces in multiple environments is challenging. The lack of focus on neighbourhood or activity space definitions in studies of the food environment is unfortunate, mainly due to the large amount of research analysing relationships between the food environment and health outcomes in which no evidence demonstrates that the neighbourhood exposures used coincide with the actual exposure. Tracking technologies can provide space-time data on the behaviour of individuals, and these data can be used to define neighbourhoods for measuring exposure to the food environment.

## Supplementary Files

Supplementary File 1
