# Supplementary material for: Defining Neighbourhoods as a Measure of Exposure to the Food Environment
Source: Int J Environ Res Public Health. 2015 Jul 21;12(7):8504–25. doi: 10.3390/ijerph120708504 (PMC4515733; doi:10.3390/ijerph120708504)
Supplement: Supplementary File 1 [file ijerph-12-08504-s001.pdf]

Supplementary Information

Defining Neighbourhoods as a Measure of Exposure to the Food Environment

Table S1. Results Tukey's HSD test for comparison of area size.

Neighbourhood		Neighbourhood	Mean Diff.	Sig.	95% Conf. Interval of the Differences	
					Lower	Upper
Parish	**	Address buffer 800 m	15.797	0.005	2.713	28.861
		Address buffer 1 mile	9.661	0.379	−3.413	22.735
	**	School buffer 800 m	15.787	0.005	2.713	28.861
		School buffer 1 mile	9.661	0.379	−3.413	22.735
	*	Address & school buffer 800 m	13.886	0.026	0.812	26.961
		Address & school buffer 1 mile	2.688	1.000	−10.386	15.762
	**	Convex hull	−33.337	<0.000	−46.411	−20.262
		1 standard deviational ellipses	0.020	1.000	−13.054	13.094
	**	2 standard deviational ellipses	−33.592	<0.000	−46.667	−20.518
		Path area	13.042	0.051	−0.032	26.116
Address buffer 800 m		Address buffer 1 mile	−6.126	0.916	−19.200	6.948
		School buffer 800 m			Exactly same area size	
		School buffer 1 mile	−6.126	0.917	−19.200	6.948
		Address & school buffer 800 m	−1.900	1.000	−14.974	11.174
	*	Address & school buffer 1 mile	−13.098	0.049	−26.173	−0.024
	**	Convex hull	−49.123	<0.000	−62.197	−36.049
	**	1 standard deviational ellipses	−15.766	0.005	−28.840	−2.692
	**	2 standard deviational ellipses	49.379	<0.000	−62.453	−36.305
		Path area	−2.745	1.000	−15.819	10.329
Address buffer 1 mile		School buffer 800 m	6.126	0.917	−6.948	19.200
		School buffer 1 mile			Exactly same area size	
		Address & school buffer 800 m	4.226	0.994	−8.848	17.300
		Address & school buffer 1 mile	−6.972	0.826	−20.045	6.101
	**	Convex hull	−42.997	<0.000	−56.071	−29.923
		1 standard deviational ellipses	−9.640	0.383	−22.714	3.434
	**	2 standard deviational ellipses	−42.253	<0.000	−56.327	−30.179
		Path area	3.381	0.999	−9.693	16.455
School buffer 800 m		School buffer 1 mile	−6.126	0.917	−19.200	6.948
		Address & school buffer 800 m	−1.900	1.000	−14.974	11.174
	*	Address & school buffer 1 mile	−13.098	0.049	−26.173	−0.024
	**	Convex hull	−49.123	<0.000	−62.197	−36.049
	**	1 standard deviational ellipses	−15.766	0.005	−28.840	−2.692
	**	2 standard deviational ellipses	−49.379	<0.000	−62.453	−36.305
		Path area	−2.745	1.000	−15.819	10.329

Table S1. *Cont.*

Neighbourhood		Neighbourhood	Mean Diff.	Sig.	95% Conf. Interval of the Differences	
					Lower	Upper
School buffer 1 mile		Address & school buffer 800 m	4.226	0.994	−8.848	17.300
		Address & school buffer 1 mile	−6.972	0.826	−20.046	6.102
	**	Convex hull	−42.997	<0.000	−56.071	−29.923
		1 standard deviational ellipses	−9.640	0.383	−22.714	3.434
	**	2 standard deviational ellipses	−43.253	<0.000	−56.327	−30.179
		Path area	3.381	0.999	−9.693	16.455
Address & school buffer 800 m		Address & school buffer 1 mile	−11.198	0.174	−24.272	1.876
	**	Convex hull	−47.223	<0.000	−60.297	−34.149
	*	1 standard deviational ellipses	−13.866	0.027	−26.940	−0.792
	**	2 standard deviational ellipses	−47.479	<0.000	−60.553	−34.405
		Path area	−0.845	1.000	−13.919	12.230
Address & school buffer 1 mile	**	Convex hull	−36.025	<0.000	−49.099	−22.951
		1 standard deviational ellipses	−2.668	1.000	−15.742	10.406
	**	2 standard deviational ellipses	−36.281	<0.000	−49.355	−23.207
		Path area	10.354	0.275	−2.720	23.428
Convex hull	**	1 standard deviational ellipses	33.357	<0.000	20.283	46.431
		2 standard deviational ellipses	−0.256	1.000	−13.330	12.818
	**	Path area	46.379	<0.000	33.304	59.453
1 standard deviational ellipses	**	2 standard deviational ellipses	−33.613	<0.000	−46.687	−20.539
		Path area	13.022	0.052	−0.053	26.096
2 standard deviational ellipses	**	Path area	46.634	<0.000	33.560	59.709

Notes: * Statistically significant below the 0.05 level, ** Statistically significant below the 0.01 level.

Table S2. Results Tukey's HSD test for comparison of area size in urban sample.

Neighbourhood		Neighbourhood	Mean Diff.	Sig.	95% Conf. Interval of the Differences	
					Lower	Upper
Parish		Address buffer 800 m	3.691	0.644	−2.240	9.621
		Address buffer 1 mile	−2.435	0.965	−8.366	3.495
		School buffer 800 m	3.691	0.644	−2.240	9.621
		School buffer 1 mile	−2.435	0.965	−8.366	3.495
		Address & school buffer 800 m	1.900	0.994	−4.031	7.830
	**	Address & school buffer 1 mile	−8.291	<0.000	−14.222	−2.360
	**	Convex hull	−15.439	<0.000	−21.370	−9.509
		1 standard deviational ellipses	1.176	1.000	−4.754	7.107
	**	2 standard deviational ellipses	−1.099	<0.000	−16.920	−5.059
		Path area	2.252	0.980	−3.679	8.182
Address buffer 800 m	*	Address buffer 1 mile	−6.126	0.036	−12.057	−0.195
		School buffer 800 m			Exactly same area size	
	*	School buffer 1 mile	−6.126	0.036	−12.057	−0.195
		Address & school buffer 800 m	−1.791	0.997	−7.722	4.140
	**	Address & school buffer 1 mile	−11.982	<0.000	−17.912	−6.051
	**	Convex hull	−19.130	<0.000	−25.061	−13.199
		1 standard deviational ellipses	−2.515	0.956	−8.445	3.416
	**	2 standard deviational ellipses	−14.680	<0.000	−20.611	−8.749
		Path area	−1.439	0.999	−7.370	4.491

Table S2. *Cont.*

Neighbourhood		Neighbourhood	Mean Diff.	Sig.	95% Conf. Interval of the Differences	
					Lower	Upper
Address buffer 1 mile	*	School buffer 800 m	6.126	0.036	0.195	12.057
		School buffer 1 mile			Exactly same area size	
		Address & school buffer 800 m	4.335	0.395	−1.596	10.266
		Address & school buffer 1 mile	−5.856	0.056	−11.786	0.075
	**	Convex hull	−13.004	<0.000	−18.934	−7.073
		1 standard deviational ellipses	3.612	0.674	−2.319	9.542
	**	2 standard deviational ellipses	−8.554	<0.000	−14.485	−2.623
		Path area	4.687	0.277	−1.244	10.618
School buffer 800 m	*	School buffer 1 mile	−6.126	0.036	−12.057	−0.195
		Address & school buffer 800 m	−1.791	0.997	−7.722	4.140
	**	Address & school buffer 1 mile	−11.982	<0.000	−17.912	−6.051
	**	Convex hull	−19.130	<0.000	−25.061	−13.199
		1 standard deviational ellipses	−2.515	0.956	−8.445	3.416
	**	2 standard deviational ellipses	−14.680	<0.000	−20.611	−8.749
		Path area	−1.439	0.999	−7.370	4.491
School buffer 1 mile		Address & school buffer 800 m	4.335	0.395	−1.596	10.266
		Address & school buffer 1 mile	−5.856	0.056	−11.786	0.075
	**	Convex hull	−13.004	<0.000	−18.934	−7.073
		1 standard deviational ellipses	3.612	0.674	−2.319	9.542
	**	2 standard deviational ellipses	−8.554	<0.000	−14.485	−2.623
		Path area	4.687	0.277	−1.244	10.618
Address & school buffer 800 m	**	Address & school buffer 1 mile	−10.191	<0.000	−16.121	−4.260
	**	Convex hull	−17.339	<0.000	−23.269	−11.408
		1 standard deviational ellipses	−0.723	1.000	−6.654	5.207
	**	2 standard deviational ellipses	−12.889	<0.000	−18.820	−6.958
		Path area	0.352	1.000	−5.579	6.283
Address & school buffer 1 mile	**	Convex hull	−7.148	0.005	−13.079	−1.218
	**	1 standard deviational ellipses	9.467	<0.000	3.537	15.398
		2 standard deviational ellipses	−2.698	0.930	−8.629	3.232
	**	Path area	10.543	<0.000	4.612	16.473
Convex hull	**	1 standard deviational ellipses	16.615	<0.000	10.685	22.546
		2 standard deviational ellipses	4.450	0.354	−1.481	10.380
	**	Path area	17.691	<0.000	11.760	23.621
1 standard deviational ellipses	**	2 standard deviational ellipses	−12.166	<0.000	−18.096	−6.235
		Path area	1.075	1.000	−4.855	7.006
2 standard deviational ellipses	**	Path area	13.241	<0.000	7.310	19.172

Notes: * Statistically significant below the 0.05 level, ** Statistically significant below the 0.01 level.

Table S3. Results Tukey's HSD test for comparison of area size in rural sample.

Neighbourhood		Neighbourhood	Mean Diff.	Sig.	95% Conf. Interval of the Differences	
					Lower	Upper
Parish	**	Address buffer 800 m	28.013	0.007	4.369	51.657
		Address buffer 1 mile	21.887	0.099	−1.757	45.531
	**	School buffer 800 m	28.013	0.007	4.369	51.657
		School buffer 1 mile	21.887	0.099	−1.757	45.531
	*	Address & school buffer 800 m	26.002	0.018	2.358	49.646
		Address & school buffer 1 mile	13.786	0.730	−9.858	37.430
	**	Convex hull	−51.426	<0.000	−75.071	−27.782
		1 standard deviational ellipses	−1.148	1.000	−24.792	22.496
	**	2 standard deviational ellipses	−5.644	<0.000	−80.083	−32.794
	*	Path area	23.948	0.044	0.304	47.592
Address buffer 800 m		Address buffer 1 mile	−6.126	0.999	−29.770	17.518
		School buffer 800 m			Exactly same area size	
		School buffer 1 mile	−6.126	0.999	−29.770	17.518
		Address & school buffer 800 m	2.011	1.000	−21.633	25.655
		Address & school buffer 1 mile	−14.227	0.690	−37.871	9.417
	**	Convex hull	−79.439	<0.000	−103.083	−55.795
	**	1 standard deviational ellipses	−29.161	0.003	−52.805	−5.517
	**	2 standard deviational ellipses	−84.451	<0.000	−108.095	−60.807
		Path area	−4.064	1.000	−27.708	19.580
Address buffer 1 mile		School buffer 800 m	6.126	0.999	−17.518	29.770
		School buffer 1 mile			Exactly same area size	
		Address & school buffer 800 m	4.115	1.000	−19.529	−27.760
		Address & school buffer 1 mile	−8.101	0.991	−31.745	15.543
	**	Convex hull	−73.331	<0.000	−96.957	−49.669
		1 standard deviational ellipses	−23.035	0.064	−46.679	0.610
	**	2 standard deviational ellipses	−78.325	<0.000	−101.969	−54.681
		Path area	2.062	1.000	−21.582	25.706
School buffer 800 m		School buffer 1 mile	−6.126	0.999	−29.770	17.518
		Address & school buffer 800 m	−2.011	1.000	−25.655	21.633
		Address & school buffer 1 mile	−14.227	0.690	−37.871	9.417
	**	Convex hull	−79.439	<0.000	−103.083	−55.795
	**	1 standard deviational ellipses	−29.161	0.004	−52.805	−5.517
	**	2 standard deviational ellipses	−84.451	<0.000	−108.095	−60.807
		Path area	−4.064	1.000	−27.708	19.580
School buffer 1 mile		Address & school buffer 800 m	4.115	1.000	−19.529	27.760
		Address & school buffer 1 mile	−8.101	0.991	−31.745	15.543
	**	Convex hull	−73.313	<0.000	−96.957	−49.669
		1 standard deviational ellipses	−23.035	0.064	−46.679	0.610
	**	2 standard deviational ellipses	−78.325	<0.000	−101.969	−54.681
		Path area	2.062	1.000	−21.582	25.706
Address & school buffer 800 m		Address & school buffer 1 mile	−12.217	0.852	−35.861	11.427
	**	Convex hull	−77.429	<0.000	−101.073	−53.785
	**	1 standard deviational ellipses	−27.150	0.010	−50.794	−3.506
	**	2 standard deviational ellipses	−82.441	<0.000	−106.085	−58.797
		Path area	−2.054	1.000	−25.698	21.590

Table S3. *Cont.*

Neighbourhood		Neighbourhood	Mean Diff.	Sig.	95% Conf. Interval of the Differences	
					Lower	Upper
Address & school buffer 1 mile	**	Convex hull	−65.212	<0.000	−88.856	−41.568
		1 standard deviational ellipses	−14.933	0.622	−38.577	8.711
	**	2 standard deviational ellipses	−70.224	<0.000	−93.868	−46.580
		Path area	10.163	0.952	−13.481	33.807
Convex hull	**	1 standard deviational ellipses	50.279	<0.000	26.635	73.923
		2 standard deviational ellipses	−5.012	1.000	−28.656	18.632
	**	Path area	75.375	<0.000	51.731	99.019
1 standard deviational ellipses	**	2 standard deviational ellipses	−55.291	<0.000	−78.935	−31.647
	*	Path area	25.096	0.027	1.452	48.740
2 standard deviational ellipses	**	Path area	80.387	<0.000	56.743	104.031

Notes: * Statistically significant below the 0.05 level, ** Statistically significant below the 0.01 level.

Table S4. Results Tukey's HSD test for comparison of percent of GPS activity within neighbourhoods.

Neighbourhood		Neighbourhood	Mean Diff.	Sig.	95% Conf. Interval of the Differences	
					Lower	Upper
Parish		Address buffer 800 m	1.051	0.999	−4.559	6.663
		Address buffer 1 mile	−2.322	0.963	−7.933	3.289
	**	School buffer 800 m	56.891	<0.000	51.280	62.502
	**	School buffer 1 mile	48.484	<0.000	42.872	54.095
	**	Address & school buffer 800 m	−10.733	<0.000	−16.345	−5.122
	**	Address & school buffer 1 mile	−14.844	<0.000	−20.455	−9.232
	**	Convex hull	−26.020	<0.000	−31.631	−20.409
	**	1 standard deviational ellipses	−7.58	0.001	−13.270	−2.047
	**	2 standard deviational ellipses	−20.374	<0.000	−25.985	−14.762
	**	Path area	−25.474	<0.000	−31.086	−19.863
Address buffer 800 m		Address buffer 1 mile	−3.374	0.692	−8.985	2.237
	**	School buffer 800 m	55.839	<0.000	50.228	61.451
	**	School buffer 1 mile	47.432	<0.000	41.820	53.043
	**	Address & school buffer 800 m	−11.785	<0.000	−17.397	−6.174
	**	Address & school buffer 1 mile	−15.895	<0.000	−21.507	−10.284
	**	Convex hull	−27.072	<0.000	−32,683	−21.461
	**	1 standard deviational ellipses	−8.710	<0.000	−14.322	−3.099
	**	2 standard deviational ellipses	−21.425	<0.000	−27.037	−15.814
	**	Path area	−26.526	<0.000	−32.138	−20.915
Address buffer 1 mile	**	School buffer 800 m	59.213	<0.000	53.602	64.825
	**	School buffer 1 mile	50.806	<0.000	45.195	56.417
	**	Address & school buffer 800 m	−8.411	<0.000	−14.023	−2.800
	**	Address & school buffer 1 mile	−12.521	<0.000	−18.133	−6.910
	**	Convex hull	−23.698	<0.000	−29.309	−18.087
		1 standard deviational ellipses	−5.336	0.079	−10.948	0.275
	**	2 standard deviational ellipses	−18.051	<0.000	−23.663	−12.440
	**	Path area	−23.152	<0.000	−28.763	−17.541

Table S4. *Cont.*

Neighbourhood		Neighbourhood	Mean Diff.	Sig.	95% Conf. Interval of the Differences	
					Lower	Upper
School buffer 800 m	**	School buffer 1 mile	−8.407	<0.000	−14.018	−2.796
	**	Address & school buffer 800 m	−67.625	<0.000	−73.236	−62.014
	**	Address & school buffer 1 mile	−71.735	<0.000	−77.346	−66.124
	**	Convex hull	−82.912	<0.000	−88,523	−77.300
	**	1 standard deviational ellipses	−64.550	<0.000	−70.161	−58.938
	**	2 standard deviational ellipses	−77.265	<0.000	−82.876	−71.654
	**	Path area	−82.366	<0.000	−87.977	−76.754
School buffer 1 mile	**	Address & school buffer 800 m	−59.217	<0.000	−64.829	−53.606
	**	Address & school buffer 1 mile	−63.328	<0.000	−68.939	−57.716
	**	Convex hull	−74.504	<0.000	−80.116	−68.893
	**	1 standard deviational ellipses	−56.142	<0.000	−61.754	−50.531
	**	2 standard deviational ellipses	−68.858	<0.000	−74.469	−63.246
	**	Path area	−73.958	<0.000	−79.57	−68.347
Address & school buffer 800 m		Address & school buffer 1 mile	−4.110	0.393	−9.721	1.501
	**	Convex hull	−15.286	<0.000	−20.898	−9.675
		1 standard deviational ellipses	3.075	0.800	−2.536	8.686
	**	2 standard deviational ellipses	−9.640	<0.000	−15.251	−4.028
	**	Path area	−14.741	<0.000	−20.352	9.129
Address & school buffer 1 mile	**	Convex hull	−11.177	<0.000	−16.788	−5.565
	**	1 standard deviational ellipses	7.185	0.002	1.573	12.796
		2 standard deviational ellipses	−5.530	0.058	−11.141	0.081
	**	Path area	−10.630	<0.000	−16.242	−5.019
Convex hull	**	1 standard deviational ellipses	18.361	<0.000	12.750	23.973
	*	2 standard deviational ellipses	5.646	0.046	0.035	11.258
		Path area	−	1.000	−	−
1 standard deviational ellipses	**	2 standard deviational ellipses	−12.715	<0.000	−18.326	−7.103
	**	Path area	−17.815	<0.000	−23.427	−12.204
2 standard deviational ellipses	*	Path area	−5.646	0.046	−11.258	−0.035

Notes: * Statistically significant below the 0.05 level, ** Statistically significant below the 0.01 level.

Table S5. Results Tukey's HSD test for comparison of supermarket exposure.

Neighbourhood		Neighbourhood	Mean diff.	Sig.	95% Conf. Interval of the Differences	
					Lower	Upper
Parish		Address buffer 800 m	1.246	0.960	−1.735	4.227
		Address buffer 1 mile	−2.578	0.164	−5.559	0.404
		School buffer 800 m	−1.503	0.872	−4.484	1.479
	**	School buffer 1 mile	−9.219	<0.000	−12.201	−6.238
	*	Address & school buffer 800 m	−3.358	0.013	−6.340	−0.377
	**	Address & school buffer 1 mile	−13.267	<0.000	−16.249	−10.286
	**	Convex hull	−23.011	<0.000	−25.992	−20.029
		1 standard deviational ellipses	−2.936	0.058	−5.917	0.046
	**	2 standard deviational ellipses	−16.610	<0.000	−19.591	−13.628
	**	Path area	−8.011	<0.000	−10.992	−5.029

Table S5. *Cont.*

Neighbourhood		Neighbourhood	Mean diff.	Sig.	95% Conf. Interval of the Differences	
					Lower	Upper
Address buffer 800 m	**	Address buffer 1 mile	−3.824	0.002	−6.805	−0.842
		School buffer 800 m	−2.749	0.103	−5.730	0.233
	**	School buffer 1 mile	−10.465	<0.000	−13.447	−7.484
	**	Address & school buffer 800 m	−4.604	<0.000	−7.586	−1.623
	**	Address & school buffer 1 mile	−14.513	<0.000	−17.495	−11.532
	**	Convex hull	−24.257	<0.000	−27.238	−21.275
	**	1 standard deviational ellipses	−4.182	<0.000	−7.163	−1.200
	**	2 standard deviational ellipses	−17.856	<0.000	−20.837	−14.874
	**	Path area	−9.257	<0.000	−12.238	−6.275
Address buffer 1 mile		School buffer 800 m	1.075	0.986	−1.907	4.056
	**	School buffer 1 mile	−6.642	<0.000	−9.623	−3.660
		Address & school buffer 800 m	−0.781	0.999	−3.762	2.201
	**	Address & school buffer 1 mile	−10.690	<0.000	−13.671	−7.708
	**	Convex hull	−20.433	<0.000	−23.415	−17.452
		1 standard deviational ellipses	−0.358	1.000	−3.340	2.623
	**	2 standard deviational ellipses	−14.032	<0.000	−17.013	−11.051
	**	Path area	−5.433	<0.000	−8.415	−2.452
School buffer 800 m	**	School buffer 1 mile	−7.717	<0.000	−10.698	−4.735
		Address & school buffer 800 m	−1.856	0.645	−4.837	1.126
	**	Address & school buffer 1 mile	−11.765	<0.000	−14.746	−8.783
	**	Convex hull	−21.508	<0.000	−24.489	−18.527
		1 standard deviational ellipses	−1.433	0.903	−4.415	1.548
	**	2 standard deviational ellipses	−15.107	<0.000	−18.088	−12.126
	**	Path area	−6.508	<0.000	−9.489	−3.527
School buffer 1 mile	**	Address & school buffer 800 m	5.861	<0.000	2.880	8.842
	**	Address & school buffer 1 mile	−4.048	0.001	−7.030	−1.067
	**	Convex hull	−13.791	<0.000	−16.773	−10.810
	**	1 standard deviational ellipses	6.283	<0.000	3.302	9.265
	**	2 standard deviational ellipses	−7.390	<0.000	−10.372	−4.409
		Path area	1.209	0.968	−1.773	4.190
Address & school buffer 800 m	**	Address & school buffer 1 mile	−9.909	<0.000	−12.890	−6.928
	**	Convex hull	−19.652	<0.000	−22.634	−163.671
		1 standard deviational ellipses	0.422	1.000	−2.559	3.404
	**	2 standard deviational ellipses	−13.251	<0.000	−16.233	−10.270
	**	Path area	−4.652	<0.000	−7.634	−1.671
Address & school buffer 1 mile	**	Convex hull	−9.743	<0.000	−12.725	−6.762
	**	1 standard deviational ellipses	10.332	<0.000	7.350	13.313
	*	2 standard deviational ellipses	−3.342	0.014	−6.324	−0.361
	**	Path area	5.257	<0.000	2.275	8.238
Convex hull	**	1 standard deviational ellipses	20.075	<0.000	17.093	23.056
	**	2 standard deviational ellipses	6.401	<0.000	3.420	9.382
	**	Path area	15.000	<0.000	12.019	17.981
1 standard deviational ellipses	**	2 standard deviational ellipses	−13.674	<0.000	−16.655	−10.692
	**	Path area	−5.075	<0.000	−8.056	−2.093
2 standard deviational ellipses	**	Path area	8.599	<0.000	5.618	11.580

Notes: * Statistically significant below the 0.05 level, ** Statistically significant below the 0.01 level.

Table S6. Results Tukey's HSD test for comparison of supermarket exposure in urban sample.

Neighbourhood		Neighbourhood	Mean Diff.	Sig.	95% Conf. Interval of the Differences	
					Lower	Upper
Parish**		Address buffer 800 m	1.138	0.996	−2.64	4.940
	**	Address buffer 1 mile	−5.500	<0.000	−9.302	−1.697
		School buffer 800 m	−0.117	1.000	−3.919	3.685
	**	School buffer 1 mile	−7.648	<0.000	−11.450	−3.846
		Address & school buffer 800 m	−2.968	0.294	−6.770	0.833
	**	Address & school buffer 1 mile	−13.968	<0.000	−17.770	−10.166
	**	Convex hull	−17.585	<0.000	−21.387	−13.783
		1 standard deviational ellipses	−1.244	0.993	−5.046	2.557
	**	2 standard deviational ellipses	−11.510	<0.000	−15.312	−7.708
	**	Path area	−5.776	<0.000	−9.578	−1.974
Address buffer 800 m	**	Address buffer 1 mile	−6.638	<0.000	−10.440	−2.836
		School buffer 800 m	−1.255	0.993	−5.057	2.546
	**	School buffer 1 mile	−8.787	<0.000	−12.589	−4.985
	*	Address & school buffer 800 m	−4.106	0.022	−7.908	−0.304
	**	Address & school buffer 1 mile	−15.106	<0.000	−18.908	−11.304
	**	Convex hull	−18.723	<0.000	−22.525	−14.921
		1 standard deviational ellipses	−2.382	0.634	−6.185	1.419
	**	2 standard deviational ellipses	−12.648	<0.000	−16.450	−8.846
	**	Path area	−6.914	<0.000	−10.716	−3.112
Address buffer 1 mile	**	School buffer 800 m	5.382	<0.000	1.580	9.185
		School buffer 1 mile	−2.148	0.766	−5.950	1.653
		Address & school buffer 800 m	2.531	0.542	−1.270	6.333
	**	Address & school buffer 1 mile	−8.468	<0.000	−12−270	−4.666
	**	Convex hull	−12.085	<0.000	−15.887	−8.283
	*	1 standard deviational ellipses	4.255	0.015	0.453	8.057
	**	2 standard deviational ellipses	−.010	<0.000	−9.812	−2.208
		Path area	−0.276	1.000	−4.078	3.525
School buffer 800 m	**	School buffer 1 mile	−7.531	<0.000	−11.531	−3.729
		Address & school buffer 800 m	−2.851	0.355	−6.653	0.950
	**	Address & school buffer 1 mile	−13.851	<0.000	−17.653	−10.049
	**	Convex hull	−17.468	<0.000	−21.270	−13.666
		1 standard deviational ellipses	−1.127	0.997	−4.929	2.674
	**	2 standard deviational ellipses	−11.393	<0.000	−15.195	−7.591
	**	Path area	−5.659	<0.000	−9.461	−1.857
School buffer 1 mile	**	Address & school buffer 800 m	4.680	0.004	0.878	8.482
	**	Address & school buffer 1 mile	−6.319	<0.000	−10.121	−2.517
	**	Convex hull	−9.936	<0.000	−13.738	−6.134
	**	1 standard deviational ellipses	6.404	<0.000	2.602	10.206
	*	2 standard deviational ellipses	−3.861	0.043	−7.663	−0.059
		Path area	1.872	0.887	−1.926	5.674
Address & school buffer 800 m	**	Address & school buffer 1 mile	−11.000	<0.000	−14.802	−7.197
	**	Convex hull	−14.617	<0.000	−18.419	−10.815
		1 standard deviational ellipses	1.723	0.931	−2.078	5.525
	**	2 standard deviational ellipses	−8.542	<0.000	−12.344	−4.740
		Path area	−2.808	0.378	−6.610	0.993

Table S6. *Cont.*

Neighbourhood		Neighbourhood	Mean Diff.	Sig.	95% Conf. Interval of the Differences	
					Lower	Upper
Address & school buffer 1 mile		Convex hull	−3.617	0.079	−7.419	0.185
	**	1 standard deviational ellipses	12.723	<0.000	8.921	16.524
		2 standard deviational ellipses	2.457	0.588	−1.344	6.259
	**	Path area	8.191	<0.000	4.389	11.993
Convex hull	**	1 standard deviational ellipses	16.340	<0.000	12.538	20.340
	**	2 standard deviational ellipses	6.074	<0.000	2.272	9.876
	**	Path area	11.808	<0.000	8.006	15.611
1 standard deviational ellipses	**	2 standard deviational ellipses	−10.265	<0.000	−14.067	−6.463
	**	Path area	−4.531	0.006	−8.333	−0.729
2 standard deviational ellipses	**	Path area	5.734	<0.000	1.932	9.536

Notes: * Statistically significant below the 0.05 level, ** Statistically significant below the 0.01 level.

Table S7. Results Tukey's HSD test for comparison of supermarket exposure in rural sample.

Neighbourhood		Neighbourhood	Mean Diff.	Sig.	95% Conf. Interval of the Differences	
					Lower	Upper
Parish		Address buffer 800 m	1.354	0.995	−3.002	5.712
		Address buffer 1 mile	0.376	1.000	−3.980	4.733
		School buffer 800 m	−2.903	0.542	−7.260	1.453
	**	School buffer 1 mile	−10.806	<0.000	−15.163	−6.449
		Address & school buffer 800 m	−3.752	0.168	−8.109	0.604
	**	Address & school buffer 1 mile	−12.559	<0.000	−16.916	−8.201
	**	Convex hull	−28.494	<0.000	−32.815	−24.137
	*	1 standard deviational ellipses	−4.645	0.025	−9.002	−0.288
	**	2 standard deviational ellipses	−21.763	<0.000	−26.120	−17.406
	**	Path area	−10.268	<0.000	−14.625	−5.911
Address buffer 800 m		Address buffer 1 mile	−0.978	0.999	−5.335	3.378
		School buffer 800 m	−4.258	0.062	−8.615	0.099
	**	School buffer 1 mile	−12.161	<0.000	−16.518	−7.804
	**	Address & school buffer 800 m	−5.107	0.008	−9.464	−0.750
	**	Address & school buffer 1 mile	−13.913	<0.000	−18.271	−9.556
	**	Convex hull	−29.849	<0.000	−34.206	−25.492
	**	1 standard deviational ellipses	−6.000	0.001	−10.357	−1.642
	**	2 standard deviational ellipses	−23.118	<0.000	−27.475	−18.761
	**	Path area	−11.623	<0.000	−15.980	−7.266
Address buffer 1 mile		School buffer 800 m	−3.279	0.349	−7.636	1.077
	**	School buffer 1 mile	−11.182	<0.000	−15.539	−6.825
		Address & school buffer 800 m	−4.129	0.082	−8.486	0.228
	**	Address & school buffer 1 mile	−12.934	<0.000	−17.292	−8.578
	**	Convex hull	−28.871	<0.000	−33.228	−24.513
	**	1 standard deviational ellipses	−5.021	0.009	−9.378	−0.664
	**	2 standard deviational ellipses	−22.139	<0.000	−26.496	−17.782
	**	Path area	−10.645	<0.000	−15.002	−6.287

Table S7. *Cont.*

Neighbourhood		Neighbourhood	Mean Diff.	Sig.	95% Conf. Interval of the Differences	
					Lower	Upper
School buffer 800 m	**	School buffer 1 mile	−7.903	<0.000	−12.260	−3.546
		Address & school buffer 800 m	−0.849	0.999	−5.206	3.507
	**	Address & school buffer 1 mile	−9.655	<0.000	−14.013	−5.298
	**	Convex hull	−25.591	<0.000	−29.948	−21.234
		1 standard deviational ellipses	−1.741	0.971	−6.099	2.615
	**	2 standard deviational ellipses	−18.860	<0.000	−23.217	−14.503
	**	Path area	−7.365	<0.000	−11.722	−3.008
School buffer 1 mile	**	Address & school buffer 800 m	7.053	<0.000	2.696	11.410
		Address & school buffer 1 mile	−1.752	0.969	−6.109	2.604
	**	Convex hull	−17.688	<0.000	−22.045	−13.330
	**	1 standard deviational ellipses	6.161	<0.000	1.804	10.518
	**	2 standard deviational ellipses	−10.956	<0.000	−15.314	−6.599
		Path area	0.537	0.999	−3.819	4.895
Address & school buffer 800 m	**	Address & school buffer 1 mile	−8.806	<0.000	−13.163	−4.449
	**	Convex hull	−24.741	<0.000	−29.099	−20.384
		1 standard deviational ellipses	−0.892	0.999	−5.249	3.464
	**	2 standard deviational ellipses	−18.010	<0.000	−22.367	−13.653
	**	Path area	−6.516	<0.000	−10.873	−2.158
Address & school buffer 1 mile	**	Convex hull	−15.935	<0.000	−20.292	−11.578
	**	1 standard deviational ellipses	7.913	<0.000	3.556	12.271
	**	2 standard deviational ellipses	−9.204	<0.000	−13.561	−4.847
		Path area	2.290	0.837	−2.066	6.647
Convex hull	**	1 standard deviational ellipses	23.849	<0.000	19.492	28.206
	**	2 standard deviational ellipses	6.731	<0.000	2.374	11.088
	**	Path area	18.225	<0.000	13.868	22.582
1 standard deviational ellipses	**	2 standard deviational ellipses	−17.118	<0.000	−21.475	−12.761
	**	Path area	−5.623	0.002	−9.980	−1.266
2 standard deviational ellipses	**	Path area	11.494	<0.000	7.137	15.851

Notes: * Statistically significant below the 0.05 level, ** Statistically significant below the 0.01 level.

Table S8. Results Tukey's HSD test for comparison of supermarket per square kilometre exposure in urban sample.

Neighbourhood		Neighbourhood	Mean Diff.	Sig.	95% Conf. Interval of the Differences	
					Lower	Upper
Parish		Address buffer 800 m	−0.264	0.958	−0.893	0.365
		Address buffer 1 mile	0.230	0.984	−0.398	0.860
	**	School buffer 800 m	−0.888	<0.000	−1.517	−0.259
		School buffer 1 mile	−0.033	1.000	−0.662	0.595
		Address & school buffer 800 m	−0.543	0.165	−1.172	0.086
		Address & school buffer 1 mile	0.140	0.999	−0.488	0.770
		Convex hull	−0.429	0.503	−1.059	0.199
		1 standard deviational ellipses	−0.431	0.497	−1.061	0.197
		2 standard deviational ellipses	−0.002	1.000	−0631	0.627
	**	Path area	−1.915	<0.000	−2.545	−1.286

Table S8. *Cont.*

Neighbourhood		Neighbourhood	Mean Diff.	Sig.	95% Conf. Interval of the Differences	
					Lower	Upper
Address buffer 800 m		Address buffer 1 mile	0.494	0.284	−0.134	1.124
		School buffer 800 m	−0.624	0.054	−1.253	0.005
		School buffer 1 mile	0.230	0.984	−0.398	0.860
		Address & school buffer 800 m	−0.278	0.940	−0.908	0.350
		Address & school buffer 1 mile	0.405	0.594	−0.224	1.034
		Convex hull	−0.165	0.998	−0.795	0.463
		1 standard deviational ellipses	−0.167	0.998	−0.796	0.462
		2 standard deviational ellipses	0.262	0.960	−0.367	0.891
	**	Path area	−1.651	<0.000	−2.280	−1.022
Address buffer 1 mile	**	School buffer 800 m	−1.119	<0.000	−1.748	−0.489
		School buffer 1 mile	−0.264	0.958	−0.893	0.365
	**	Address & school buffer 800 m	−0.773	0.004	−1.403	−0.144
		Address & school buffer 1 mile	−0.089	0.999	−0.719	0.539
	*	Convex hull	−0.660	0.030	−1.289	−0.031
	*	1 standard deviational ellipses	−0.662	0.029	−1.291	−0.032
		2 standard deviational ellipses	−0.232	0.983	−0.861	0.396
	**	Path area	−2.145	<0.000	−2.775	−1.516
School buffer 800 m	**	School buffer 1 mile	0.855	0.001	0.225	1.484
		Address & school buffer 800 m	0.345	0.797	−0.283	0.974
	**	Address & school buffer 1 mile	1.029	<0.000	0.399	1.658
		Convex hull	0.458	0.399	−0.171	1.087
		1 standard deviational ellipses	0.456	0.405	−0.172	1.086
	**	2 standard deviational ellipses	0.886	<0.000	0.257	1.515
	**	Path area	−1.027	<0.000	−1.656	−0.397
School buffer 1 mile		Address & school buffer 800 m	−0.509	0.243	−1.138	0.119
		Address & school buffer 1 mile	0.174	0.998	−0.455	0.803
		Convex hull	−0.39	0.626	−1.025	0.232
		1 standard deviational ellipses	−0.398	0.621	−1.027	0.231
		2 standard deviational ellipses	0.031	1.000	−0.597	0.660
	**	Path area	−1.882	<0.000	−2.511	−1.252
Address & school buffer 800 m	*	Address & school buffer 1 mile	0.684	0.020	0.054	1.313
		Convex hull	0.113	0.999	−0.516	0.742
		1 standard deviational ellipses	0.111	0.999	−0.517	0.740
		2 standard deviational ellipses	0.541	0.169	−0.088	1.170
	**	Path area	−1.372	<0.000	−2.002	−0.743
Address & school buffer 1 mile		Convex hull	−0.571	0.116	−1.200	0.058
		1 standard deviational ellipses	−0.572	0.113	−1.201	0.057
		2 standard deviational ellipses	−0.142	0.999	−0.772	0.486
	**	Path area	−2.056	<0.000	−2.685	−1.427
Convex hull		1 standard deviational ellipses	−0.002	1.000	−0.631	0.627
		2 standard deviational ellipses	0.427	0.510	−0.201	1.057
	**	Path area	−1.485	<0.000	−2.115	−0.856
1 standard deviational ellipses		2 standard deviational ellipses	0.429	0.504	−0.199	1.058
	**	Path area	−1.484	<0.000	−2.113	−0.854
2 standard deviational ellipses	**	Path area	−1.913	<0.000	−2.543	−1.284

Notes: * Statistically significant below the 0.05 level, ** Statistically significant below the 0.01 level.

Table S9. Results Tukey's HSD test for comparison of supermarket per square kilometre exposure in rural sample.

Neighbourhood		Neighbourhood	Mean Diff.	Sig.	95% Conf. Interval of the Differences	
					Lower	Upper
Parish		Address buffer 800 m	−0.325	0.054	−0.653	0.003
		Address buffer 1 mile	−0.127	0.975	−0.455	0.200
	**	School buffer 800 m	.2.443	<0.000	−2.771	−2.115
	**	School buffer 1 mile	−1.501	<0.000	−1.829	−1.173
	**	Address & school buffer 800 m	−1.384	<0.000	−1.712	−1.056
	**	Address & school buffer 1 mile	−0.811	<0.000	−.1.139	−0.483
	**	Convex hull	−0.638	<0.000	−0.966	−0.310
	*	1 standard deviational ellipses	−0.343	0.030	−0.671	0.016
	*	2 standard deviational ellipses	−0.351	0.025	−0.679	−0.023
	**	Path area	−2.142	<0.000	−2.470	−1.814
Address buffer 800 m		Address buffer 1 mile	0.197	0.686	−0.130	0.525
	**	School buffer 800 m	−2.117	<0.000	−2.445	−1.789
	**	School buffer 1 mile	−1.176	<0.000	−1.504	−0.848
	**	Address & school buffer 800 m	1.058	<0.000	−1.386	−0.730
	**	Address & school buffer 1 mile	−0.486	<0.000	−0.814	−0.158
		Convex hull	−0.312	0.077	−0.640	0.015
		1 standard deviational ellipses	−0.018	1.000	−0.346	0.309
		2 standard deviational ellipses	−0.025	1.000	−0.355	0.302
	**	Path area	−1.817	<0.000	−2.145	−1.489
Address buffer 1 mile	**	School buffer 800 m	−2.315	<0.000	−2.643	−1.987
	**	School buffer 1 mile	−1.374	<0.000	−1.702	−1.046
	**	Address & school buffer 800 m	−1.256	<0.000	−1.584	−0.928
	**	Address & school buffer 1 mile	−0.684	<0.000	−1.013	−0.356
	**	Convex hull	−0.510	<0.000	−0.838	−0.182
		1 standard deviational ellipses	−0.216	0.558	−0.544	0.111
		2 standard deviational ellipses	−0.223	0.509	−0.551	0.104
	**	Path area	−2.014	<0.000	−2.342	−1.686
School buffer 800 m	**	School buffer 1 mile	0.941	<0.000	0.613	1.269
	**	Address & school buffer 800 m	1.058	<0.000	0,731	1.386
	**	Address & school buffer 1 mile	1.631	<0.000	1.303	1.959
	**	Convex hull	1.804	<0.000	1.476	2.133
	**	1 standard deviational ellipses	2.099	<0.000	1.771	2.427
	**	2 standard deviational ellipses	2.092	<0.000	1.764	2.420
		Path area	0.300	0.107	−0027	0.628
School buffer 1 mile		Address & school buffer 800 m	0.117	0.986	−0.210	0.445
	**	Address & school buffer 1 mile	0.690	<0.000	0.361	1.018
	**	Convex hull	0.863	<0.000	0.535	1.191
	**	1 standard deviational ellipses	1.158	<0.000	0.830	1.486
	**	2 standard deviational ellipses	1.151	<0.000	0.823	1.479
	**	Path area	−0.640	<0.000	−0.968	−0.312

Table S9. *Cont.*

Neighbourhood		Neighbourhood	Mean Diff.	Sig.	95% Conf. Interval of the Differences	
					Lower	Upper
Address & school buffer 800 m	**	Address & school buffer 1 mile	0.572	<0.000	0.244	0.900
	**	Convex hull	0.746	<0.000	0.418	1.074
	**	1 standard deviational ellipses	1.040	<0.000	0.712	1.368
	**	2 standard deviational ellipses	1.033	<0.000	0.705	1.361
	**	Path area	−0.758	<0.000	−1.086	−0.430
Address & school buffer 1 mile		Convex hull	0.173	0.831	−0.154	0.501
	**	1 standard deviational ellipses	0.468	<0.000	0.140	0.795
	**	2 standard deviational ellipses	0.461	<0.000	0.133	0.789
	**	Path area	−1.330	<0.000	−1.658	−1.002
Convex hull		1 standard deviational ellipses	0.294	0.125	−0.033	0.622
		2 standard deviational ellipses	0.287	0.148	−0.040	0.615
	**	Path area	−1.504	<0.000	−1.832	−1.176
1 standard deviational ellipses		2 standard deviational ellipses	−0.007	1.000	−0.334	0.321
	**	Path area	−1.798	<0.000	−2.126	−1.470
2 standard deviational ellipses	**	Path area	−1.791	<0.000	−2.119	−1.463

Notes: * Statistically significant below the 0.05 level, ** Statistically significant below the 0.01 level.

Table S10. Results Tukey's HSD test for comparison of fast food outlet exposure.

Neighbourhood		Neighbourhood	Mean Diff.	Sig.	95% Conf. Interval of the Differences	
					Lower	Upper
Parish		Address buffer 800 m	0.251	1.000	−5.770	6.273
		Address buffer 1 mile	−5.722	0.080	−11.743	0.299
	**	School buffer 800 m	−9.642	<0.000	−15.663	−3.621
	**	School buffer 1 mile	−22.925	<0.000	−28.946	−16.904
	**	Address & school buffer 800 m	−12.406	<0.000	−18.428	−6.385
	**	Address & school buffer 1 mile	−29.011	<0.000	−35.032	−22.990
	**	Convex hull	−42.856	<0.000	−48.877	−36.834
	**	1 standard deviational ellipses	−7.241	0.005	−13.262	−1.219
	**	2 standard deviational ellipses	−30.920	<0.000	−36.941	−24.899
	**	Path area	−20.225	<0.000	−26.246	−14.203
Address buffer 800 m		Address buffer 1 mile	−5.973	0.054	−11.994	0.048
	**	School buffer 800 m	−9.893	<0.000	−15.914	−3.872
	**	School buffer 1 mile	−23.176	<0.000	−29.198	−17.155
	**	Address & school buffer 800 m	−12.658	<0.000	−18.679	−6.637
	**	Address & school buffer 1 mile	−29.262	<0.000	−35.283	−23.241
	**	Convex hull	−43.107	<0.000	−49.128	−37.086
	**	1 standard deviational ellipses	−7.492	0.003	−13.513	−1.471
	**	2 standard deviational ellipses	−31.171	<0.000	−37.192	−25.150
	**	Path area	−20.476	<0.000	−26.497	−14.455

Table S10. *Cont.*

Neighbourhood		Neighbourhood	Mean Diff.	Sig.	95% Conf. Interval of the Differences	
					Lower	Upper
Address buffer 1 mile		School buffer 800 m	−3.920	0.579	−9.941	2.101
	**	School buffer 1 mile	−17.203	<0.000	−23.224	−11.182
	*	Address & school buffer 800 m	−6.684	0.016	−12.706	−0.663
	**	Address & school buffer 1 mile	−23.289	<0.000	−29.310	−17.268
	**	Convex hull	−37.134	<0.000	−43.155	−31.112
		1 standard deviational ellipses	−1.519	0.999	−7.540	4.502
	**	2 standard deviational ellipses	−25.198	<0.000	−31.219	−19.177
	**	Path area	−14.503	<0.000	−20.524	−8.481
School buffer 800 m	**	School buffer 1 mile	−13.283	<0.000	−19.305	−7.262
		Address & school buffer 800 m	−2.765	0.927	−8.786	3.256
	**	Address & school buffer 1 mile	−19.369	<0.000	−25.390	−13.348
	**	Convex hull	−33.214	<0.000	−39.235	−27.193
		1 standard deviational ellipses	2.401	0.971	−3.620	8.422
	**	2 standard deviational ellipses	−21.278	<0.000	−27.299	−15.257
	**	Path area	−10.583	<0.000	−16.604	−4.562
School buffer 1 mile	**	Address & school buffer 800 m	10.519	<0.000	4.498	16.540
	*	Address & school buffer 1 mile	−6.086	0.045	−12.107	−0.064
	**	Convex hull	−19.930	<0.000	−25.952	−13.909
	**	1 standard deviational ellipses	15.684	<0.000	9.663	21.706
	**	2 standard deviational ellipses	−7.995	0.001	−14.016	−1.973
		Path area	2.701	0.937	−3.321	8.722
Address & school buffer 800 m	**	Address & school buffer 1 mile	−16.604	<0.000	−22.625	−10.583
	**	Convex hull	−30.449	<0.000	−36.470	−24.428
		1 standard deviational ellipses	5.166	0.173	−0.855	11.187
	**	2 standard deviational ellipses	−18.513	<0.000	−24.535	−12.492
	**	Path area	−7.818	0.001	−13.839	−1.797
Address & school buffer 1 mile	**	Convex hull	−13.845	<0.000	−19.866	−7.824
	**	1 standard deviational ellipses	21.770	<0.000	15.749	27.791
		2 standard deviational ellipses	−1.909	0.995	−7.930	4.112
	**	Path area	8.786	<0.000	2.765	14.807
Convex hull	**	1 standard deviational ellipses	35.615	<0.000	29.594	41.636
	**	2 standard deviational ellipses	11.936	<0.000	5.915	17.957
	**	Path area	22.631	<0.000	16.610	28.652
1 standard deviational ellipses	**	2 standard deviational ellipses	−23.679	<0.000	−29.700	−17.658
	**	Path area	−12.984	<0.000	−19.005	−6.963
2 standard deviational ellipses	**	Path area	10.695	<0.000	4.674	16.716

Notes: * Statistically significant below the 0.05 level, ** Statistically significant below the 0.01 level.

Table S11. Results Tukey's HSD test for comparison of fast food outlet exposure in urban sample.

Neighbourhood		Neighbourhood	Mean Diff.	Sig.	95% Conf. Interval of the Differences	
					Lower	Upper
Parish		Address buffer 800 m	−0.414	1.000	−8.710	7.880
	**	Address buffer 1 mile	−11.372	0.001	−19.667	−3.077
		School buffer 800 m	−6.829	0.222	−15.124	1.465
	**	School buffer 1 mile	−19.627	<0.000	−27.922	−11.332
	**	Address & school buffer 800 m	−11.595	<0.000	−19.890	−3.300
	**	Address & school buffer 1 mile	−30.106	<0.000	−38.401	−21.811
	**	Convex hull	−36.000	<0.000	−44.295	−27.704
		1 standard deviational ellipses	−5.308	0.603	−13.603	2.986
	**	2 standard deviational ellipses	−24.659	<0.000	−32.954	−16.364
	**	Path area	−17.159	<0.000	−25.454	−8.864
Address buffer 800 m	**	Address buffer 1 mile	−10.957	0.001	−19.252	−2.662
		School buffer 800 m	−6.414	0.308	−14.710	1.880
	**	School buffer 1 mile	−19.212	<0.000	−27.507	−10.917
	**	Address & school buffer 800 m	−11.180	0.001	−19.476	−2.885
	**	Address & school buffer 1 mile	−29.691	<0.000	−37.986	−21.396
	**	Convex hull	−35.585	<0.000	−43.880	−27.289
		1 standard deviational ellipses	−4.893	0.715	−13.188	3.401
	**	2 standard deviational ellipses	−24.244	<0.000	−32.539	−15.949
	**	Path area	−16.744	<0.000	−25.039	−8.449
Address buffer 1 mile		School buffer 800 m	4.542	0.799	−3.752	12.837
		School buffer 1 mile	−8.255	0.052	−16.550	0.039
		Address & school buffer 800 m	−0.223	1.000	−8.518	8.071
	**	Address & school buffer 1 mile	−18.734	<0.000	−27.029	−10.438
	**	Convex hull	−24.627	<0.000	−32.992	−16.332
		1 standard deviational ellipses	6.063	0.394	−2.231	14.359
	**	2 standard deviational ellipses	−13.287	<0.000	−21.582	−4.992
		Path area	−5.787	0.469	−14.082	−2.507
School buffer 800 m	**	School buffer 1 mile	−12.797	<0.000	−21.093	−4.502
		Address & school buffer 800 m	−4.765	0.747	−13.061	3.529
	**	Address & school buffer 1 mile	−23.276	<0.000	−31.571	−14.981
	**	Convex hull	−29.170	<0.000	−37.465	−20.875
		1 standard deviational ellipses	1.521	0.999	−6.773	9.816
	**	2 standard deviational ellipses	−17.829	<0.000	−26.124	−9.534
	**	Path area	−10.329	0.003	−18.624	−2.034
School buffer 1 mile		Address & school buffer 800 m	8.031	0.068	−0.263	16.327
	**	Address & school buffer 1 mile	−10.478	0.002	−18.773	−2.183
	**	Convex hull	−16.372	<0.000	−24.667	−8.077
	**	1 standard deviational ellipses	14.319	<0.000	6.023	22.614
		2 standard deviational ellipses	−5.031	0.790	−13.327	3.263
		Path area	2.468	0.996	−5.827	10.763
Address & school buffer 800 m	**	Address & school buffer 1 mile	−18.510	<0.000	−26.805	−10.215
	**	Convex hull	−24.404	<0.000	−32.699	−16.109
		1 standard deviational ellipses	6.287	0.338	−2.007	14.582
	**	2 standard deviational ellipses	−13.063	<0.000	−21.359	−4.768
		Path area	−5.563	0.531	−13.859	2.731

Table S11. *Cont.*

Neighbourhood		Neighbourhood	Mean Diff.	Sig.	95% Conf. Interval of the Differences	
					Lower	Upper
Address & school buffer 1 mile		Convex hull	−5.893	0.440	−14.188	2.401
	**	1 standard deviational ellipses	24.797	<0.000	16.502	33.093
		2 standard deviational ellipses	5.446	0.564	−2.848	13.742
	**	Path area	12.946	<0.000	4.651	21.242
Convex hull	**	1 standard deviational ellipses	30.691	<0.000	22.396	38.986
	**	2 standard deviational ellipses	11.340	<0.000	3.045	19.635
	**	Path area	18.840	<0.000	10.545	27.135
1 standard deviational ellipses	**	2 standard deviational ellipses	−19.351	<0.000	−27.646	−11.055
	**	Path area	−11.851	<0.000	−20.146	−3.555
2 standard deviational ellipses		Path area	7.500	0.119	−0.795	15.795

Note: * Statistically significant below the 0.05 level, ** Statistically significant below the 0.01 level.

Table S12. Results Tukey's HSD test for comparison of fast food outlet exposure in rural sample.

Neighbourhood		Neighbourhood	Mean Diff.	Sig.	95% Conf. Interval of the Differences	
					Lower	Upper
Parish		Address buffer 800 m	0.924	0.999	−7.558	9.407
		Address buffer 1 mile	−0.010	1.000	−8.493	8.471
	**	School buffer 800 m	−12.483	<0.000	−20.966	−4.001
	**	School buffer 1 mile	−26.258	<0.000	−34.740	−17.775
	**	Address & school buffer 800 m	−13.225	<0.000	−21.708	−4.743
	**	Address & school buffer 1 mile	−27.903	<0.000	−36.385	−19.420
	**	Convex hull	−49.784	<0.000	−58.267	−41.302
	*	1 standard deviational ellipses	−9.193	0.021	−17.676	−0.710
	**	2 standard deviational ellipses	−37.247	<0.000	−45.730	−28.764
	**	Path area	−23.322	<0.000	−31.805	−14.839
Address buffer 800 m		Address buffer 1 mile	−0.935	0.999	−9.418	7.547
	**	School buffer 800 m	−13.408	<0.000	−21.891	−4.925
	**	School buffer 1 mile	−27.182	<0.000	−35.65	−18.700
	**	Address & school buffer 800 m	−14.150	<0.000	−22.633	−5.667
	**	Address & school buffer 1 mile	−28.827	<0.000	−37.310	−20.345
	**	Convex hull	−50.709	<0.000	−59.192	−42.226
	**	1 standard deviational ellipses	−10.118	0.006	−18.601	−1.635
	**	2 standard deviational ellipses	−38.172	<0.000	−46.654	−29.689
	**	Path area	−24.247	<0.000	−32.730	−15.764
Address buffer 1 mile	**	School buffer 800 m	−12.473	<0.000	−20.955	−3.990
	**	School buffer 1 mile	−26.247	<0.000	−34.730	−17.764
	**	Address & school buffer 800 m	−13.215	<0.000	−21.697	−4.732
	**	Address & school buffer 1 mile	−27.892	<0.000	−36.275	−19.409
	**	Convex hull	−49.774	<0.000	−58.256	−41.291
	*	1 standard deviational ellipses	−9.182	0.021	−17.665	−0.700
	**	2 standard deviational ellipses	−37.236	<0.000	−45.719	−28.753
	**	Path area	−23.311	<0.000	−31.794	−14.829

Table S12. *Cont.*

Neighbourhood		Neighbourhood	Mean Diff.	Sig.	95% Conf. Interval of the Differences	
					Lower	Upper
School buffer 800 m	**	School buffer 1 mile	−13.774	<0.000	−22.256	−5.291
		Address & school buffer 800 m	−0.741	1.000	−9.224	7.740
	**	Address & school buffer 1 mile	−15.419	<0.000	−23.902	−6.936
	**	Convex hull	−37.301	<0.000	−45.783	−28.818
		1 standard deviational ellipses	3.290	0.976	−5.192	11.773
	**	2 standard deviational ellipses	−24.763	<0.000	−33.246	−16.280
	**	Path area	−10.838	0.002	−19.321	−2.355
School buffer 1 mile	**	Address & school buffer 800 m	13.032	<0.000	4.549	21.515
		Address & school buffer 1 mile	−1.645	0.999	−10.127	6.837
	**	Convex hull	−23.526	<0.000	−32.009	−15.044
	**	1 standard deviational ellipses	17.064	<0.000	8.581	25.547
	**	2 standard deviational ellipses	−10.989	0.002	−19.471	−2.506
		Path area	2.935	0.989	−5.547	11.418
Address & school buffer 800 m	**	Address & school buffer 1 mile	−14.677	<0.000	−32.160	−6.194
	**	Convex hull	−36.559	<0.000	−45.041	−28.076
		1 standard deviational ellipses	4.032	0.908	−4.450	12.515
	**	2 standard deviational ellipses	−24.021	<0.000	−32.504	−15.538
	**	Path area	−10.096	0.006	−18.579	−1.614
Address & school buffer 1 mile	**	Convex hull	−21.881	<0.000	−30.364	−13.398
	**	1 standard deviational ellipses	18.709	<0.000	10.226	27.192
	*	2 standard deviational ellipses	−9.344	0.017	−17.826	−0.861
		Path area	4.580	0.813	−3.902	13.063
Convex hull	**	1 standard deviational ellipses	40.591	<0.000	32.108	49.074
	**	2 standard deviational ellipses	12.537	<0.000	4.054	21.020
	**	Path area	26.462	<0.000	17.979	34.945
1 standard deviational ellipses	**	2 standard deviational ellipses	−28.053	<0.000	−36.536	−19.571
	**	Path area	−14.129	<0.000	−22.611	−5.646
2 standard deviational ellipses	**	Path area	13.924	<0.000	5.441	22.407

Notes: * Statistically significant below the 0.05 level, ** Statistically significant below the 0.01 level.

Table S13. Results Tukey's HSD test for comparison of fast food outlet per square kilometre exposure in urban sample.

Neighbourhood		Neighbourhood	Mean Diff.	Sig.	95% Conf. Interval of the Differences	
					Lower	Upper
Parish		Address buffer 800 m	0.195	0.999	−1.910	2.301
		Address buffer 1 mile	1.414	0.529	−0.691	3.520
	**	School buffer 800 m	−2.994	<0.000	−5.101	−0.888
		School buffer 1 mile	0.399	0.999	−1.706	2.506
		Address & school buffer 800 m	−1.239	0.718	−3.345	0.867
		Address & school buffer 1 mile	0.954	0.931	−1.151	3.060
		Convex hull	−0.796	0.980	−2.902	1.310
		1 standard deviational ellipses	−0.084	1.000	−2.190	2.021
		2 standard deviational ellipses	0.103	1.000	−2.002	2.210
	**	Path area	−4.610	<0.000	−6.716	−2.504

Table S13. *Cont.*

Neighbourhood		Neighbourhood	Mean Diff.	Sig.	95% Conf. Interval of the Differences	
					Lower	Upper
Address buffer 800 m		Address buffer 1 mile	1.218	0.738	−0.887	3−324
	**	School buffer 800 m	−3.190	<0.000	−5.296	−1.084
		School buffer 1 mile	0.204	0.999	−1.901	2.310
		Address & school buffer 800 m	−1.434	0.507	−3.541	0.671
		Address & school buffer 1 mile	0.758	0.986	−1.347	2.864
		Convex hull	−0.991	0.913	−3.097	1.114
		1 standard deviational ellipses	−0.279	0.999	−2.386	1.826
		2 standard deviational ellipses	−0.091	1.000	−2.197	2.014
	**	Path area	−4.806	<0.000	−6.912	−2.700
Address buffer 1 mile	**	School buffer 800 m	−4.409	<0.000	−6.515	−2.303
		School buffer 1 mile	−1.014	0.901	−3.120	1.091
	**	Address & school buffer 800 m	−2.653	0.003	−4.759	−0.547
		Address & school buffer 1 mile	−0.460	0.999	−2.566	1.646
	*	Convex hull	−2.210	0.030	−4.316	−0.104
		1 standard deviational ellipses	−1.498	0.437	−3.604	0.607
		2 standard deviational ellipses	−1.310	0.643	−3.416	0.795
	**	Path area	−6.024	<0.000	−8.131	−3.918
School buffer 800 m	**	School buffer 1 mile	3.394	<0.000	1.288	5.501
		Address & school buffer 800 m	1.755	0.206	−0.350	3.861
	**	Address & school buffer 1 mile	3.949	<0.000	1.843	6.055
	*	Convex hull	2.198	0.032	0.093	4.304
	**	1 standard deviational ellipses	2.910	<0.000	0.804	5.016
	**	2 standard deviational ellipses	3.098	<0.000	0.992	5.204
		Path area	−1.615	0.321	−3.721	0.490
School buffer 1 mile		Address & school buffer 800 m	−1.639	0.299	−3.745	0.467
		Address & school buffer 1 mile	0.554	0.998	−1.551	2.660
		Convex hull	−1.195	0.761	−3.302	0.910
		1 standard deviational ellipses	−0.484	0.999	−2.590	1.621
		2 standard deviational ellipses	−0.295	0.999	−2.402	1.810
	**	Path area	−5.010	<0.000	−7.116	2.904
Address & school buffer 800 m	*	Address & school buffer 1 mile	2.193	0.033	0.087	4.299
		Convex hull	0.443	0.999	−1.663	2.549
		1 standard deviational ellipses	1.154	0.798	−0.951	3.261
		2 standard deviational ellipses	1.343	0.608	−0.763	3.449
	**	Path area	−3.371	<0.000	−5.477	−1.265
Address & school buffer 1 mile		Convex hull	−1.750	0.209	−3.856	0.355
		1 standard deviational ellipses	−1.038	0.886	−3.144	1.067
		2 standard deviational ellipses	−0.850	0.968	−2.956	1.255
	**	Path area	−5.564	<0.000	−7.671	−3.458
Convex hull		1 standard deviational ellipses	0.711	0.991	−1,394	2.817
		2 standard deviational ellipses	0.899	0.953	−1.206	3.006
	**	Path area	−3.814	<0.000	−5.920	−1.708
1 standard deviational ellipses		2 standard deviational ellipses	0.188	1.000	−1.917	2.294
	**	Path area	−4.526	<0.000	−6.632	−2.420
2 standard deviational ellipses	**	Path area	−4.714	<0.000	−6.820	−2.608

Notes: * Statistically significant below the 0.05 level, ** Statistically significant below the 0.01 level.

Table S14. Results Tukey's HSD test for comparison of fast food outlet per square kilometre exposure in rural sample.

Neighbourhood		Neighbourhood	Mean Diff.	Sig.	95% Conf. Interval of the Differences	
					Lower	Upper
Parish		Address buffer 800 m	−0.278	0.999	−1.415	0.858
		Address buffer 1 mile	−0.115	0.999	−1.252	1.021
	**	School buffer 800 m	−6.947	<0.000	−8.083	−5.810
	**	School buffer 1 mile	−3.341	<0.000	−4.477	−2.204
	**	Address & school buffer 800 m	−3.612	<0.000	−4.749	−2.476
	**	Address & school buffer 1 mile	−1.729	<0.000	−2.866	−0.593
	*	Convex hull	−1.239	0.019	−2.375	−0.102
		1 standard deviational ellipses	−0.660	0.734	−1.797	0.476
		2 standard deviational ellipses	−0.639	0.771	−1.775	0.497
	**	Path area	−4.567	0	−5.703	−3.430
Address buffer 800 m		Address buffer 1 mile	0.163	0.999	−0.973	1.299
	**	School buffer 800 m	−6.668	<0.000	−7.805	−5.532
	**	School buffer 1 mile	−3.062	<0.000	−4.199	−1.926
	**	Address & school buffer 800 m	−3.334	<0.000	−4.471	−2.197
	**	Address & school buffer 1 mile	−1.451	0.002	−2.588	−0.314
		Convex hull	−0.960	0.189	−2.097	0.175
		1 standard deviational ellipses	−0.382	0.991	−1.518	0.754
		2 standard deviational ellipses	−0.361	0.994	−1.497	0.775
	**	Path area	−4.288	0	−5.425	−3.152
Address buffer 1 mile	**	School buffer 800 m	−6.831	<0.000	−7.968	−5.695
	**	School buffer 1 mile	−3.225	<0.000	−4.362	−2.089
	**	Address & school buffer 800 m	−33.497	<0.000	−4.633	2.360
	**	Address & school buffer 1 mile	−1.614	<0.000	−2.750	−0.477
		Convex hull	−1.123	0.055	−2.260	0.013
		1 standard deviational ellipses	−0.544	0.903	−1.681	0.597
		2 standard deviational ellipses	−0.523	0.924	−1.660	0.612
	**	Path area	−4.451	<0.000	−5.588	−3.315
School buffer 800 m	**	School buffer 1 mile	3.605	<0.000	2.469	4.742
	**	Address & school buffer 800 m	3.334	<0.000	2.197	4.471
	**	Address & school buffer 1 mile	5.217	<0.000	4.080	6.354
	**	Convex hull	5.708	<0.000	4.571	6.844
	**	1 standard deviational ellipses	6.286	<0.000	5.150	7.423
	**	2 standard deviational ellipses	6.307	<0.000	5.171	7.444
	**	Path area	2.380	<0.000	1.243	3.516
School buffer 1 mile		Address & school buffer 800 m	−0.271	0.999	−1.408	0.865
	**	Address & school buffer 1 mile	1.611	<0.000	0.474	2.748
	**	Convex hull	2.102	<0.000	0.965	3.238
	**	1 standard deviational ellipses	2.680	<0.000	1.544	3.817
	**	2 standard deviational ellipses	2.701	<0.000	1.565	3.838
	*	Path area	−1.225	0.022	−2.362	−0.089

Table S14. *Cont.*

Neighbourhood		Neighbourhood	Mean Diff.	Sig.	95% Conf. Interval of the Differences	
					Lower	Upper
Address & school buffer 800 m	**	Address & school buffer 1 mile	1.882	<0.000	0.746	3.019
	**	Convex hull	2.373	<0.000	1.236	3.510
	**	1 standard deviational ellipses	2.952	<0.000	1.815	4.089
	**	2 standard deviational ellipses	2.973	<0.000	1.836	4.110
		Path area	−0.954	0.197	−2.091	0.182
Address & school buffer 1 mile		Convex hull	0.490	0.950	−0.645	1.627
		1 standard deviational ellipses	1.069	0.087	−0.067	2.206
		2 standard deviational ellipses	1.090	0.073	−0.046	2.227
	**	Path area	−2.837	<0.000	−3.974	−1.701
Convex hull		1 standard deviational ellipses	0.578	0.863	−0.557	1.715
		2 standard deviational ellipses	0.599	0.834	−0.536	1.736
	**	Path area	−3.328	<0.000	−4.464	−2.191
1 standard deviational ellipses		2 standard deviational ellipses	0.021	1.000	−1.115	1.157
	**	Path area	−3.906	<0.000	−5.043	−2.770
2 standard deviational ellipses	**	Path area	−3.927	<0.000	−5.064	−2.791

Notes: * Statistically significant below the 0.05 level, ** Statistically significant below the 0.01 level.

© 2015 by the authors; licensee MDPI, Basel, Switzerland. This article is an open access article distributed under the terms and conditions of the Creative Commons Attribution license (http://creativecommons.org/licenses/by/4.0/).
